# Natural antisense transcript *Nat9a* suppresses *Scn9a* (Na_V_1.7) expression in parvalbumin-positive proprioceptive and inhibitory neurons

**DOI:** 10.1038/s41598-026-48500-8

**Published:** 2026-04-16

**Authors:** Shengnan Li, Sonia Santana-Varela, Hajar Mikaeili, Abdella M. Habib, Janvi Patel, Naxi Tian, Andrei L. Okorokov, James J. Cox

**Affiliations:** 1 Wolfson Institute for Biomedical Research, Division of Medicine, University College London, Gower Street, London, WC1E 6BT UK; 2 College of Medicine, QU Health, Qatar University, PO Box 2713, Doha, Qatar

**Keywords:** Natural antisense transcript, Long non-coding RNA, *Scn9a*, Na_V_1.7, *Scn1a*, Na_V_1.1, Pain insensitivity, Proprioception, Genetics, Molecular biology, Neuroscience

## Abstract

Natural antisense transcripts (NATs) are important spatial and temporal regulators of gene expression. We previously cloned a NAT (*Nat9a)* to *Scn9a*, which encodes the Na_V_1.7 voltage-gated sodium channel that is essential for pain perception. By studying a novel *Nat9a* global knockout mouse model, we demonstrate that the deletion of *Nat9a* does not affect pain sensitivity but instead partially impairs motor coordination. We show that *Nat9a* is expressed throughout the peripheral and central nervous systems and its expression is enriched, but not limited to, cells that express parvalbumin (*Pvalb),* which is a marker for proprioceptors in DRG and a subclass of interneurons in spinal cord and brain. *Nat9a* knockout leads to an increase in *Scn9a* expression, confirming *Nat9a*’s physiological role as a negative regulator of *Scn9a*. Furthermore, CRISPR activation of *Nat9a* transcription in Cad cells suppresses endogenous *Scn9a* expression. Notably *Nat9a* expression in *Pvalb* + neurons coincides with that of the *Scn1a* gene (Na_V_1.1 sodium channel) with both genes sharing a divergent bi-part promoter region. This suggests a mechanism by which *Nat9a* transcription leads to suppression of *Scn9a* and promotes neuronal expression of Na_V_1.1 over Na_V_1.7.

**Supplementary Information:**

The online version contains supplementary material available at 10.1038/s41598-026-48500-8.

## Introduction

Chronic pain is a major global public health issue^1–5^. Approximately one in five adults suffer from pain and another one in ten adults are diagnosed with chronic pain each year. Despite this significant clinical burden, little progress has been made in terms of therapeutic development with pain still often poorly treated and having a severe impact on the quality of life for sufferers^6^. A major reason for the poor progress in the development of new analgesics is our incomplete understanding of pain as a disease due to its complex nature. Chronic pain patients comprise a myriad of pain disorders with numerous genes and cellular pathways involved^7^.

*Scn9a* encodes a voltage-gated sodium channel (Na_V_1.7) which is expressed predominantly in dorsal root ganglia (DRG), sympathetic, olfactory sensory and hypothalamic neurons. Gain of function mutations in this sodium channel in humans cause paroxysmal extreme pain disorder (PEPD), primary erythromelalgia (IEM) and small fibre neuropathy (SFN)^8^. Recessive loss of function mutations result in a complete inability to perceive pain and anosmia^9,10^. Over the last decade, Na_V_1.7 has been a heavily studied target in the pharmaceutical industry with several selective blockers of this channel undergoing clinical trials. Despite these intense efforts, a new Na_V_1.7-based painkiller with efficacious blockade of pain, but without autonomic side effects, is unfortunately not imminent in the clinic^11–13^.

We previously reported a new regulatory gene from the *Scn9a* locus, the *Scn9*a natural antisense transcript (*Nat9a*)^14^. The *Scn9a* chromosomal region also includes *Scn1a* and *Scn7a* genes located down- and upstream respectively. We described both human and mouse *Nat9a* genes, including splice variants, and showed that *Scn9a* and *Nat9a* share overlapping exonic sequences (with 3’ overlap, tail-to-tail orientation) and that *Scn9a* and *Nat9a* are expressed in similar tissues (e.g. brain, spinal cord, DRG)^14^. We also showed that exogenous overexpression of the *Nat9a* transcript in cell line-based systems leads to downregulation of Na_V_1.7-based sodium currents^14^.

Natural antisense transcripts (NATs) are a subclass of long non-coding RNAs and are encoded on the opposite strand to their sense genes^15–21^. Approximately 70% of human genes have antisense transcripts^22^. NATs can function *in cis* (i.e. at the same locus as the sense gene) or *in trans* (at different chromosomal loci). Most NATs function *in cis*^17,18,23–27^ and more than 70% of the cis-NATs are orientated with a 3’ overlap and 15% with a 5’ overlap^22,28^. NATs are frequently expressed at a low level and in a tissue specific manner which is consistent with having specific spatial and/or developmental functions^28,29^. NATs are known as gene regulators, either promoting or inhibiting their sense gene and their function may impact on disease^30^.

Our previous data suggested that *Nat9a* and *Scn9a* form a regulatory loop with the NAT serving as a negative regulator^14^. The negative regulation by NATs can be achieved at both transcriptional and/or post-transcriptional levels, where the former includes transcriptional interference and epigenetic silencing of the sense gene promoter and the latter involving silencing via antisense encoded microRNAs, splicing interference, RNA editing and changes in subnuclear and subcellular localisation(s), all achieved via RNA-RNA homology in overlapping regions^17,19,21,31–33^ . However, a typical and simplest scenario for a negative regulatory mechanism would involve direct transcriptional interference, where active transcription complexes of both strands physically interfere with each other^21,29^.

There are important examples of previously reported neuronal antisense transcripts that play a role in regulating their respective sense gene, such as *Kcna2* NAT – the antisense for *Kcna2* which encodes for voltage-gated potassium channel subfamily A member 2^34^; *Bdnf-as –* the antisense to brain-derived neurotrophic factor^35–38^; *Penk-as*—the proenkephalin A antisense gene^39^; and others^17,29,40^.

Here, by studying a new *Nat9a* global knockout mouse model, we show that the deletion of the *Nat9a* gene does not affect the pain sensitivity of mice lacking *Nat9a*, with partially impaired motor coordination in male mice being the only detectable behavioural difference between knockout and wild type animals. We show that *Nat9a* is expressed throughout the peripheral and central neuronal systems and enriched in cells that express parvalbumin (*Pvalb),* which is a marker for proprioceptors in the PNS (DRG) and parvalbumin expressing (*Pvalb* +) interneurons in the CNS (spinal cord and brain).

*Nat9a* knockout in those cells led to an increase in *Scn9a* expression confirming *Nat9a*’s role as a negative regulator of *Scn9a*. Notably, *Nat9a* expression in *Pvalb* + neurons coincides with that of the *Scn1a* gene (Na_V_1.1 sodium channel) with both genes sharing a divergent promoter region. This suggests a mechanism in neurons that express Na_V_1.1 over Na_V_1.7 whereby the endogenous *Scn9a* expression is suppressed by *Nat9a* transcription.

## Materials and methods

### *Nat9a* knockout mouse line

The *Nat9a* knockout mouse line (Gm13629-DEL-CAS-LINE1-B6N; stock code RQ2284) was generated by MRC Harwell using a targeted CRISPR/Cas9 approach. Three heterozygous male mice, in which the *Gm13629* (*Nat9a*) gene containing a 430 bp deletion (chr2:66,271,191–66,271,620; build GRCm39/mm39) and insertion of a single nucleotide (A), were imported alongside three female wild-type C57BL/6NTac mice that were used for breeding. Mice were housed on a 12:12 h light–dark cycle with food and water available ad libitum. For genotyping, genomic DNA was isolated from ear tissue biopsies and the following PCR primers used: WT Forward 5′-AGG AAG AAT GAC CAG AAG CAT G; WT/MUT Reverse 5′- TAT TAC TCT GCA AAG CAA CCT AC; and MUT Forward 5′-CCT GAA GTG TTA ATC TGA AGA AAC. From a WT allele, the PCR product for WT Forward and WT/MUT reverse is 273 bp. There is no product amplified from the mutant allele. From a mutant allele, the PCR product for MUT Forward and WT/MUT reverse is 121 bp (due to the 430 bp deletion and an insertion of a single nucleotide).

### Behavioural tests

All animal procedures were carried out at University College London and were approved by local ethical review committees, conformed to UK Home Office regulations and the study is reported in accordance with ARRIVE guidelines (ARRIVE checklist in supplementary). Experimental groups were established through randomization procedures and behavioural assays performed blind. Adult female and male *Nat9a* knockout and wild-type littermates participated in the behavioural tests.

*Rotarod test* The motor function of mice was measured using rotarod apparatus with two sessions of prior training. Mice were individually placed on a rod with a baseline speed of 4 revolutions per minute (RPM) with an acceleration over 3 min to the maximum speed of 40 RPM. Each measurement lasted for 5 min and repeated three times per animal to calculate an average. The time spent on the apparatus, speed and distance travelled was recorded.

*Von Frey* Mice were habituated for one hour in darkened enclosures with a wire mesh floor. A series of von Frey filaments (ranging from 0.008 to 2 g) were applied to the plantar surface of the animal’s hindpaw using the up-down method and the 50% mechanical withdrawal threshold for von Frey stimulation was calculated^41^.

*Randall Selitto* Animals were restrained in an acrylic cylinder tube, and acclimatised with the sound of the running apparatus. A 3 mm^2^ blunt probe was applied to the tail of the animal with increasing pressure until the mouse exhibited a nocifensive response^42^. Three measurements were carried out for each animal with a cut-off of 500 g.

*Hargreaves’ test* Animals were acclimatised in the testing Perspex box with a glass base for at least one hour prior to the test. Radiant heat was then locally applied to the plantar surface of the hindpaw until the animal exhibited a nocifensive withdrawal response (30 s cut-off). Three recordings for each animal were taken, and the average of the three values was calculated^43^.

*Hot plate* Each animal was placed on a temperature-controlled hot plate maintained at a constant temperature of 50 °C. The latency to exhibit nociceptive behaviours, including hindpaw lifting, licking, shaking, or spontaneous jumping, was carefully monitored. The mouse was then immediately removed from the hot plate surface to prevent tissue damage.

*Cold plantar assay* Spinal reflex responses to cooling were assessed using the cold plantar test^44^. Animals were acclimatised in the testing Perspex box with a glass base for at least one hour prior to the test. Dry ice was compacted into a blunt 2 mL syringe and applied to the glass surface just below the hindpaw until the animal exhibited a nocifensive withdrawal response (30 s cut-off). Three recordings for each animal were taken, and the average of the three time values was calculated.

*Formalin test* Animals were acclimatized in perspex boxes for at least an hour before receiving an intraplantar injection of 20 μl of 5% formalin into the hindpaw. Behaviour was video recorded for 60 min, with nociceptive behaviours (licking and shaking) of the injected hindpaw counted manually over 5 min intervals for an hour.

*Complete Freund’s Adjuvant (CFA) test* After the previous establishment of von Frey baselines, mice received an intraplantar injection of 20 μl of CFA (Sigma-Aldrich). Mechanical pain thresholds were assessed on days 0, 1, 3, 5, 8, 11 and 14 post-injection using the up-down method.

### Expression analyses

Adult *Nat9a* knockout and wild-type littermates were euthanized by gradual displacement of air with CO_2_ (approximately 25% of the chamber volume per minute), followed by cervical dislocation. Total RNA was isolated from dissected DRG, spinal cord, hypothalamus, olfactory bulb, eye and hippocampal tissues using the PureLink RNA Micro Kit (Invitrogen) and then reverse transcribed according to standard protocols. *Nat9a* PCR primers (5′- GGG TTC GTG CAA TAC TGA GC and 5′-TTG ATC CTG GCT GTG GTA GC) mapping downstream of the exon 1 microdeletion and within exons 3 and 4 were used to show that the knockout line was a transcriptional null. Primers in the housekeeping *Hprt* gene (5′- GAT GAT GAA CCA GGT TAT GAC C and 5′- TTT CCA GTT TCA CTA ATG ACA C) were used as a positive control.

Quantitative real-time PCR was carried out for the dissected mouse tissues using the following probes (ThermoFisher) for *Scn9a* (Mm00450762_s1, Mm07294335_m1), *Scn1a* (Mm00450580_m1), *Nat9a* (Mm01277751_m1) and *Actb* (Mm01205647_g1). The expression level of target genes was normalized to the housekeeping β-actin gene mRNA. Relative expression levels were calculated using the 2^−ΔΔCt^ equation. All RT–PCR data are expressed as mean ± standard error of the mean (SEM) with significance indicated by *P ≤ 0.05, **P ≤ 0.01 and ***P ≤ 0.001 (two-tailed Student’s *t*-test).

### CRISPR activation

Plasmid 68495^45^ (Addgene) was modified for the transcriptional activation (CRISPRa) experiments as previously described^46^ to generate a CMV driven dSaCas9-VP64-p65-Rta (VPR) plasmid. Next, a gBlocks gene fragment (Integrated DNA Technologies) was designed to contain a synthetic poly(A) sequence, U6 promoter, guide sequence and modified guide scaffold^47^. This sequence was cloned into the EcoRI-NotI sites of the CRISPRa plasmid using In-Fusion cloning (Takara). Three guide RNAs targeting *Nat9a* regulatory regions were designed (5′-CAG TGT CAT TTG ATA CTA GCG, 5′-TTG CAC GAT GTC ATC ACC AGC and 5′-TCA CTC CAG ATG TGA TGG TTC) and tested, with the latter demonstrating the highest efficiency in upregulating *Nat9a* gene expression. Mouse catecholaminergic neuronal tumour cells (CAD, ECACC) were cultured in DMEM: HAMS F12 (1:1) with 2% glutamine and 8% FBS at 37 °C and 5% CO₂. Cells were seeded at 4.4 × 10^5^ cells per well in 6-well plates. CRISPR activation constructs were transfected into the cells using Lipofectamine 3000 following the manufacturer’s instructions. Total RNA was isolated 72 h post-transfection, and reverse transcription was performed using oligo d(T) and Superscript III first-strand synthesis system (Invitrogen) according to the manufacturer’s conditions. Quantitative real-time PCR was used to measure the expression levels of *Scn9a*, *Nat9a* and *Scn1a* relative to *Actb*.

### Chromatin immunoprecipitation

Hypothalamic tissues from 3 *Nat9a* knockout (KO) and 3 WT littermates were combined and homogenized with a Dounce tissue homogenizer. Chromatin immunoprecipitation (ChIP) assays were performed according to the manufacturer’s protocol using the Chromatrap Enzymatic ChIP-seq kit. Immunoprecipitations were performed overnight at 4 °C using antibodies against H3K4me3 (Abcam ab213224). Rabbit IgG were used as control for ChIP and mouse Positive Control Primer Set Actb-2 primers (Active Motif, 71071) and mouse Negative Control Primer Set 1 (Active Motif, 71011) were used as controls for qPCR. All ChIP experiments were performed in triplicates using three independent chromatin preparations. The immunoprecipitated DNA and the input DNA were analysed by real-time PCR using the ΔΔCt method and the following primers: *Scn9a* FWD 5′-TTTGCCCTTGGTAGAGCTGG and REV 5′- CATATCCTGGTGAGCGGTCC.

### RNAscope in situ hybridization

The mouse spinal cord, brain and DRG sections were obtained from adult wild type or *Nat9a*^*KO*^ C57BL/6 mice that were deeply anaesthetized with pentobarbital (i.p.) and transcardially perfused with heparinized saline (0.9% NaCl) followed by 25 ml of cold 4% paraformaldehyde in PBS (pH 7.4). DRGs were extracted from the lumbar area and post-fixed with the same fixative solution for 2 h at 4 °C before being embedded in cryopreservative solution (30% sucrose) overnight at 4 °C. Tissue samples were then placed in OCT blocks for posterior sectioning by cryostat. Sections (11 μm thick) were mounted onto Superfrost Plus (Fisher Scientific) slides, allowed to freeze-dry overnight at − 80 °C, for an immediate use, or were stored at − 80 °C in air-tight containers for no longer than a month for subsequent experiments.

Additionally, mouse DRG frozen sections ((MF-240-C57), 7–10 μm thick); mouse brain coronal frozen sections (MF-201 regions 5 (MF-201-05-C57) to 8 (MF-201-05-C57)), and region 12 ((MF-201-12-C57) for cerebellum, all 7–10 μm thick) were obtained commercially from Zyagen via AMS Biotechnology.

Spinal cord samples were obtained from male or female mice, between 30 and 60 days old, from either WT C57BL/6 or *Nat9a*^*KO*^ mice. To prepare sections of spinal cord from perfused mice, lumbar spinal cord segments (L3-L5) were removed and postfixed in the same fixative for 2 h at 4 °C. Lumbar spinal cord segments were dissected and cryo-sectioned to 10–11 μm, thaw-mounted onto Superfrost Plus (Fisher Scientific) slides, allowed to dry for 30 min at RT, and then stored at − 80 °C.

In situ hybridization (ISH) was performed using the RNAscope assay (Advanced Cell Diagnostics) following the protocol for fresh frozen samples for mouse cerebral cortex, mouse cerebellum and mouse DRG samples using Multiplex Fluorescence Kit v2. RNA localization was detected with either AF488 or Opal 520 (green), Opal 570 (red) and Opal 650 (far-red) fluorochrome dyes (Perkin Elmer) compared to DAPI staining (nuclei). In some cases TS-coumarin (blue, TS405) was used as an additional probe dye (Perkin Elmer) when DAPI was absent from the experiment—e.g. for *Pvalb*, Figs. 2, 3 and 4, and Supplementary Figs. 4 and 6.

ISH slides were mounted using Prolong Gold (ThermoFisher Scientific #P36930). Mouse RNAscope probes included *mmNat9a* (*Gm13629* #491941-c3); *mmScn9a* (#313341-c2 and #313341-c4); *mmNefh* (#443671 and #443671-c4); *mmScn1a* (#556181-c2); *mmNPY* (#313321); *mmPvalb* (#421931, #421931-c2 and #421931-c4); *mm Cgrp* (*mmCalca* #417961-c3) and *mmSst* (#404631).

Fluorescence was detected using a Zeiss LSM 880 Airyscan microscope. Images were taken at 10 × and 20 × magnifications with 4 × averaging. Tiles were stitched when more than one was used to image the area (scanned with 10% overlap), Airyscan processed and exported as 16-bit uncompressed tiff files for further basic editing in Adobe Lightroom Classic (Adobe) on a colour calibrated iMac (X-Rite) 5 k retina monitor. Some original colours were changed (in Zeiss ZEN software) to facilitate for easier presentation and accessibility, e.g. red-green pairing was changed to magenta-yellow. Final images were exported as jpeg files with 7200 pix on longest side at 300 ppi.

### Statistical analyses

Data were analysed using GraphPad Prism 9 (GraphPad Software, Inc), and results presented as mean ± SEM with n referring to the number of samples tested per group, as indicated in the figure legends.

### Data availability

All data are available in the main text or the Supplementary material.

## Results

### Scn9a expression is upregulated in selected Nat9a knockout mouse tissues

**Fig. 1:**
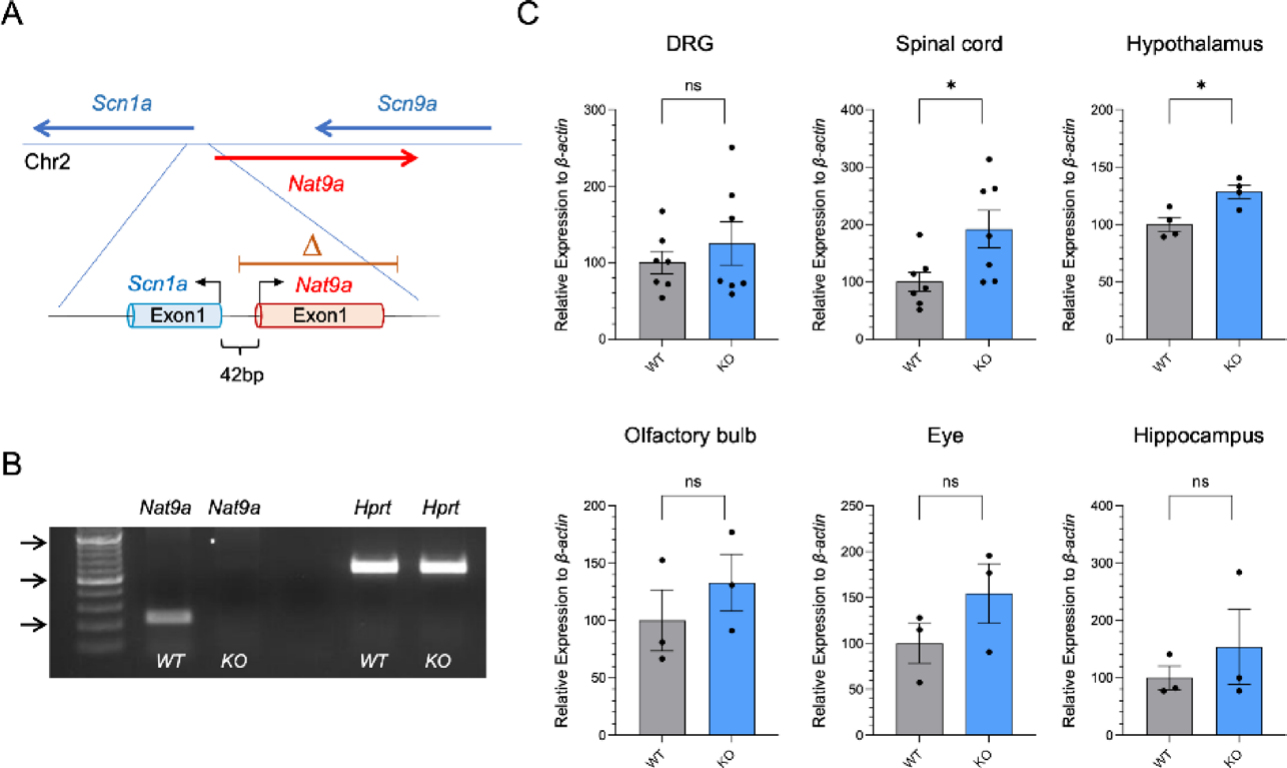
*Nat9a* CRISPR-Cas9 deletion leads to *Scn9a* mRNA expression level increase. (**A**) The 430 bp deletion site mapped in relation to surrounding exons. The arrows indicate the transcription direction. (**B**) Gel image of RT-PCR amplification of DRG cDNA from *Nat9a* knockout and wild type littermate control. *Nat9a* is amplified in the wild type mouse and no transcript is amplified from knockout mouse DRG. Housekeeping gene *Hprt* was tested in both genotypes to confirm cDNA was present in the sample. Arrows correspond to 200 bp (bottom), 500 bp (middle) and 1000 bp (top). (**C**) *Scn9a* mRNA levels rise in *Nat9a*^*KO*^ mouse (KO) when compared to wild type (WT) littermate controls as determined by qPCR in (***i***) DRG (n = 7); (***ii***) Spinal cord (n = 7, *p = 0.028; (***iii***) Hypothalamus (n = 4, *p = 0.015); (***iv***) Olfactory bulb (n = 3); (***v***) Eye (n = 3) and (***vi***) Hippocampus (n = 3). Data-points are denoted by dots, bars show the ± SEM, and data analysed by a Student’s *t*-test.

We studied a novel global knockout mouse model of *Nat9a* (Gm13629-DEL-CAS-LINE1-B6N; MRC Harwell) to better understand the function of this natural antisense transcript. The mouse model was generated using a CRISPR/Cas9 approach whereby single guide RNAs were designed to flank the first exon of the *Nat9a* gene with the aim of removing the first exon and transcriptional start site (Fig. 1A). The founder that was selected to take forward had a 430 bp deletion (chr2:66,271,191-66,271,620; build GRCm39/mm39) and insertion of a single nucleotide (A) (Fig. 1A). This male was bred to C57BL/6N females to confirm germline transmission and then heterozygous offspring were used for further breeding.

The microdeletion of exon 1 was predicted to result in no transcript being generated in the homozygous knockout mice. To test this, PCR primers were designed in exon 3 and exon 4 of *Nat9a*. PCR was performed using DRG cDNA from wild type and homozygous knockout mice. As predicted, there was a single amplicon generated from wild type DRG but no transcript amplified from knockout DRG (Fig. 1B), which was confirmed by sequencing.

Previous analysis showed a discordant expression pattern between *Scn9a* and *Nat9a*, with the sense and antisense genes often showing high *versus* low expression in a particular tissue and vice versa^14^. To check if knockout of the *Nat9a* gene resulted in changes of *Scn9a* expression levels, different mouse tissues were selected based on where *Scn9a* or *Nat9a* are endogenously highly expressed, and levels of *Scn9a* mRNA were analysed by qPCR. The results showed that knockout of *Nat9a* leads to a significant upregulation of *Scn9a* mRNA in spinal cord (*p* = 0.028) and hypothalamus (*p* = 0.015) compared to the wild type littermates (Fig. 1C). In other tissues such as DRG, olfactory bulb, eye and hippocampus, there was a trend of *Scn9a* mRNA level being also upregulated in the *Nat9a* knockouts (Fig. 1C). The statistical significance level is likely to be related to the percentage of cells in each tissue that normally express *Nat9a*, with tissues with higher levels of *Nat9a* seeing more obvious and detectable upregulation in *Scn9a* expression in the *Nat9a* knockouts.

To confirm that *Nat9a* knockout did not disrupt expression of *Scn1a*, which is located just 42 bp upstream of the *Nat9a* transcriptional start site, we checked levels of *Scn1a* mRNA from different tissues from *Nat9a* knockout and wild type littermate controls. In all selected tissues tested—DRG, spinal cord, hypothalamus, eye and hippocampus—there was no significant difference in *Scn1a* transcript levels between the knockouts and wild types (Supplementary Fig. 1) suggesting the *Nat9a* knockout model does not affect the expression of *Scn1a* and has a specific effect only on *Scn9a* levels.

### Pain sensitivity is normal in Nat9a knockouts

We next conducted acute pain behavioural tests using *Nat9a* knockout mice. Responses to mechanical stimuli were tested using von Frey filaments and the Randall Selitto test. *Nat9a* knockouts showed normal responses to light touch and to noxious mechanical stimuli (Supplementary Fig. 2A). Furthermore, responses to cold-inducing and heat-inducing noxious stimuli did not reveal any significant difference in comparison with littermate controls (Supplementary Fig. 2A). These results were consistent with behaviour tests where male and female mice were analysed separately (Supplementary Fig. 2B).

We followed this with two inflammatory models which were carried out to assess whether the absence of *Nat9a* leads to any differences in inflammatory pain responses. Injection of formalin into the hindpaw was used to induce acute inflammatory pain with the effect lasting for about an hour. In both the first and second phases of the formalin test, the results showed that there were no significant differences in the pain behavioural responses for *Nat9a* knockouts compared to their littermate controls (Supplementary Fig. 3A).

We also used CFA injections into the hindpaw that cause longer lasting hyperalgesia^48^. Withdrawal latency to mechanical stimuli was measured using the up-down von Frey method at 4 h post injection and on days 1, 3, 5, 8, 11 and 14 (Supplementary Fig. 3B and C). Again, no significant differences were observed between knockout and wild type groups.

### Nat9a is expressed in parvalbumin positive DRG proprioceptors

The apparent lack of difference in nociceptive responses between wild type and *Nat9a*^*KO*^ mice prompted us to consider that even though both *Scn9a* and *Nat9a* genes are expressed in similar tissues, such as DRG, they might be expressed in different neuronal populations within a tissue. If only a small proportion of cells express *Nat9a* in DRG, and/or *Nat9a* is expressed in cells other than nociceptors, then the knockout may not have an effect on nociception. To test this, we analysed DRG tissues from wild type mice using fluorescence in situ hybridization (FISH) technology—i.e. the RNAscope assay with confocal microscopy. To ensure the specific detection of *Scn9a* and *Nat9a* transcripts, we used probes that targeted different regions of each transcript.

**Fig. 2:**
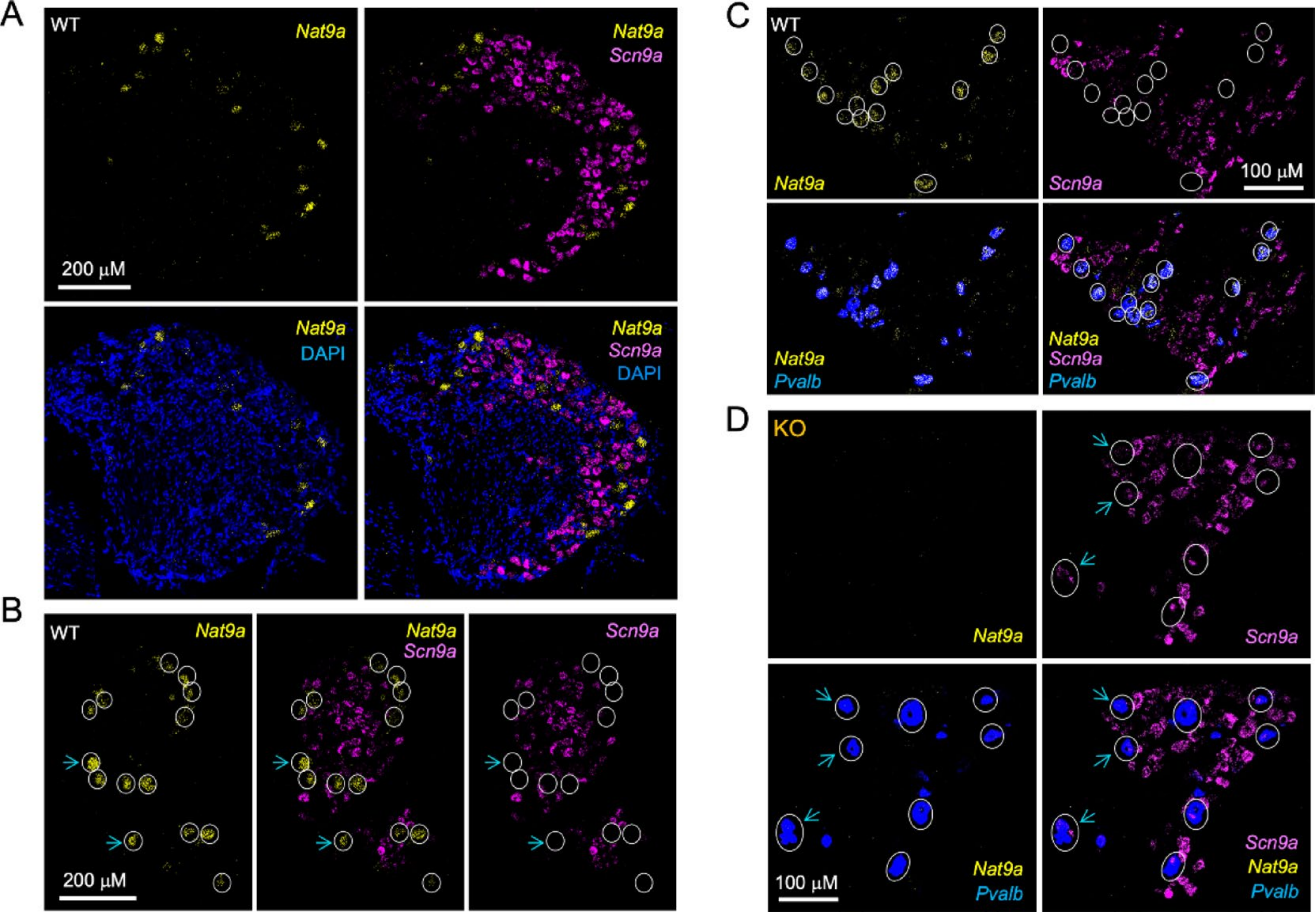
*Nat9a* lncRNA and *Scn9a* mRNA are expressed in different DRG neurons. (**A** and **B**) *Nat9a* and *Scn9a* RNA expression levels and localization in mouse DRG cells. (**A**) Cuts of fresh frozen wild type mouse DRG tissue (~ 10 μM thick) were analysed by RNAscope assay. Localization of *Scn9a* mRNA (magenta) was compared to *Nat9a* lncRNA (yellow) localization and DAPI staining indicating nuclei positions (blue). (**B**) *Nat9a* and *Scn9a* RNA expression is discordant, that is *Scn9a* (magenta) is widely expressed whereas *Nat9a* lncRNA (yellow) is detected in a small number of large neurons that typically lack *Scn9a* expression—encircled in white and a few additionally marked by cyan arrows. (**C**) *Nat9a* is expressed in *Pvalb* + neurons (proprioceptors). Cuts of fresh frozen wild type mouse DRG tissue (~ 10 μM thick) were analysed by RNAscope assay. Localization of *Nat9a* lncRNA (yellow) was compared to *Scn9a* mRNA (magenta) and *Pvalb* mRNA (blue) localization. Positions of *Pvalb* + neurons are shown by white circles and demonstrate that *Nat9a* and *Pvalb* are expressed in the same neuronal cells. (**D**) *Nat9a* knockout leads to a rise in *Scn9a* expression in mouse DRG *Pvalb* + cells. Cuts of fresh frozen *Nat9*^*KO*^ mouse (female) DRG node sections (~ 10 μM thick) were analysed by RNAscope assay. Localization of *Scn9a* mRNA (magenta) was compared to *Pvalb* mRNA (blue) localization. Positions of *Pvalb* + neurons are shown by white circles and a few additionally marked by cyan arrows and demonstrate that *Nat9a* knockout leads to *Scn9a* expression in *Pvalb* + cells. Probe for *Nat9a* lncRNA (yellow) shows no signal in *Nat9a*^*KO*^ mouse DRG. Scale bars are in white. Similar data for the male *Nat9*^*KO*^ mouse DRG are shown in Supplementary Fig. 4A.

The simultaneous visualization of *Scn9a* and *Nat9a* transcripts in fresh-frozen (FF) mouse DRG samples provided direct evidence that *Scn9a* mRNA and the *Nat9a* long non-coding RNA (lncRNA) were typically not expressed within the same neuronal cells (Fig. 2A and B). Whilst *Scn9a* mRNA was widely expressed and in most DRG neurons, *Nat9a* lncRNA was only expressed in a small amount of large sized neurons. The two genes were expressed in a largely discordant manner, that is cells expressing *Nat9a* typically did not express *Scn9a* or expressed a low level of *Scn9a* and vice versa (Fig. 2A and B, Supplementary Fig. 4D). We reasoned that the large neurons expressing *Nat9a* could be parvalbumin-positive proprioceptors as suggested by their numbers and data available from single cell RNAseq for mouse DRG^49,50^. Indeed *Nat9a* + neurons were *Pvalb* + as confirmed by RNAscope (Fig. 2C). Analysis of single cell RNAseq data^50^ confirm that (a) *Nat9a* expression is enriched in a subset of *Pvalb* + neurons and (b) across the DRG *Scn9a* is predominantly expressed in non-*Pvalb* + neurons although a subset of *Pvalb* + neurons do still express *Scn9a*, albeit at a lower level (Supplementary Fig. 5A–E).

We next tested if the removal of a sense-antisense regulatory loop in *Nat9a*^*KO*^ mice DRG *Pvalb* + cells led to a rise in *Scn9a* expression, as suggested by our qPCR data in Fig. 1C. Indeed, *Pvalb* + neurons started to ectopically express *Scn9a* mRNA in *Nat9a*^*KO*^ DRG (Fig. 2D). This was consistent for both female (Fig. 2D) and male DRG tissues (Supplementary Fig. 4A). Similarly, a discordant expression pattern in DRG was observed for *Scn1a* and *Scn9a* mRNAs, with *Scn1a* expression mostly coinciding with that of *Nat9a* (Supplementary Fig. 4B).

### *Nat9a* is highly expressed in spinal cord and *Nat9a*^***KO***^ mice show impaired motor coordination

**Fig. 3:**
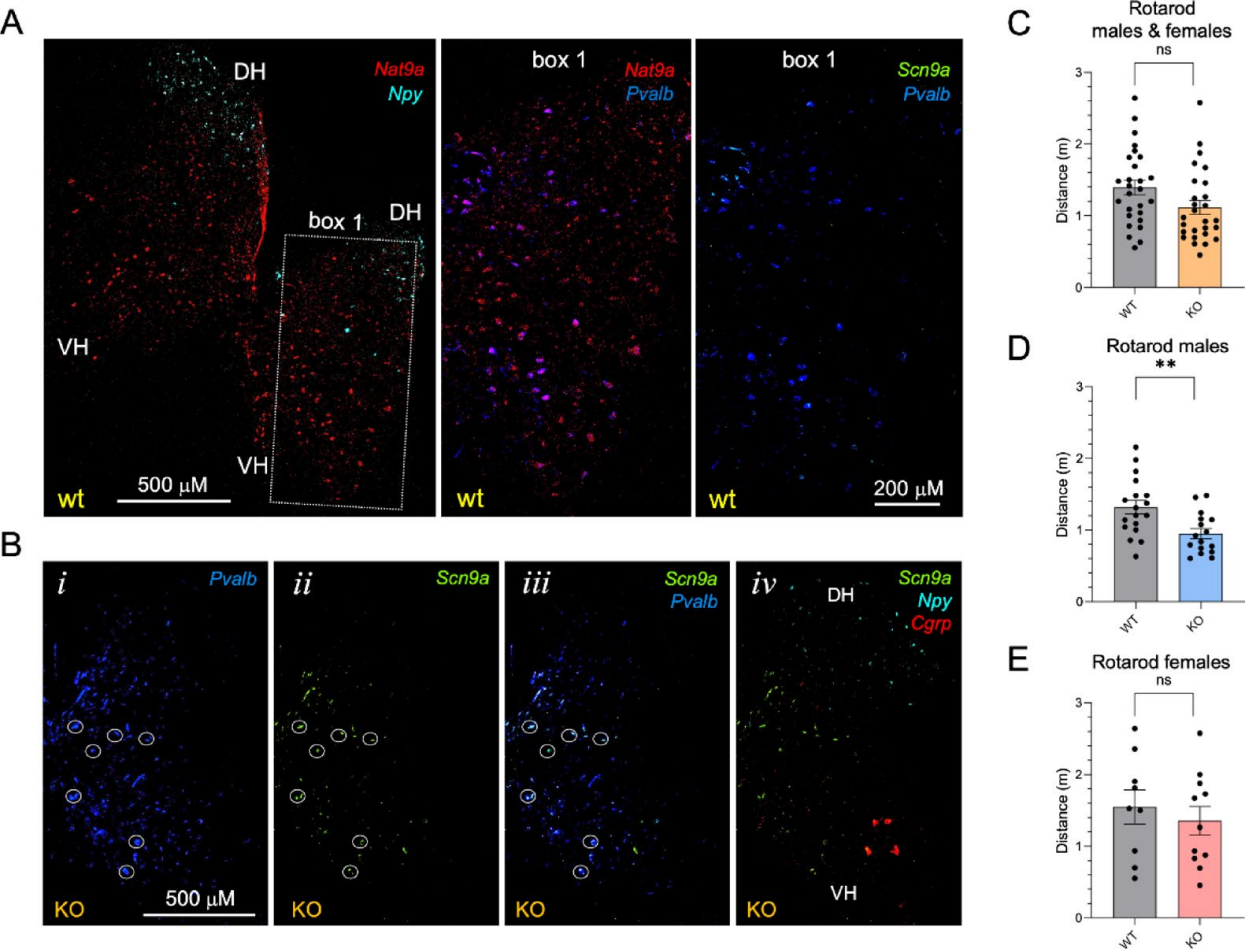
*Nat9a* is expressed in *Pvalb* + neurons in the spinal cord (**A**) Cuts of fresh frozen wild type female mouse spinal cord sections (~ 10 μM thick) were analysed by RNAscope assay. Localization of *Nat9a* lncRNA (red) was compared to *Scn9a* mRNA (green) and *Pvalb* mRNA (blue); dorsal horn areas are indicated by *Npy* mRNA expressing neurons (cyan). *Nat9a* is expressed at a high level in multiple large-sized neurons, many of which (but not all) are *Pvalb* + (Box 1). *Scn9a*, similar to in DRGs, is expressed at a very low/undetectable level in *Pvalb* + cells. Similar data for *Pvalb* and *Nat9a* and co-localisation is shown for a male mouse in Suppl. Figure 5. (**B**) *Nat9a* knockout leads to a rise in *Scn9a* expression in mouse spinal cord *Pvalb* + cells. Sections of fresh frozen *Nat9a*^*KO*^ mouse (female) spinal cord tissue (~ 10 μM thick) were analysed by RNAscope assay. (***i-iii***) Localization of *Pvalb* mRNA (blue) was compared to *Scn9a* mRNA (green) localization. (***iv***) Dorsal horn areas are indicated by *Npy* mRNA expressing neurons (cyan) and ventral horns by *Cgrp* mRNA (red). Positions of *Pvalb* + neurons are shown by white circles and demonstrate that *Nat9a* knockout leads to *Scn9a* expression in *Pvalb* + cells. Scale bars are in white. (**C**) Rotarod test in male and female *Nat9a*^*KO*^ mice and littermate controls. Motor co-ordination deficit observed in male **(D)** (**p = 0.0048) but not female **(E)** *Nat9a*^*KO*^ mice. Male WT = 18, male KO = 16, female WT = 9, female KO = 11. Data-points are denoted by dots, bars show the ± SEM, and data analysed by a Student’s *t*-test.

RNAscope analyses of wild type and *Nat9a*^*KO*^ mice spinal cord sections demonstrated that there was a large number of neurons expressing *Nat9a* lncRNA in the wild type tissue enriched in central and ventral horn areas (Fig. 3A). This was consistent with our previous data on high *Nat9a* expression levels detected in spinal cord^14^.

All *Pvalb* + interneurons in spinal cord were expressing *Nat9a* lncRNA but not *Scn9a* mRNA (Fig. 3A and Supplementary Fig. 6) and similar to DRG and brain tissues there was an increase in *Scn9a* expression in *Pvalb* + neurons from *Nat9a*^*KO*^ mice spinal cord samples (Fig. 3B). There were also high expression levels of *Nat9a* lncRNA in motor neurons which prompted us to test whether *Nat9a*^*KO*^ mice have any differences in motor function.

To that end we used the rotarod test which evaluates the motor coordination of the animal**.** The total distance travelled by *Nat9a* knockout and wild type littermates was not statistically different when data from males and females was combined, initially suggesting these mice have normal motor learning skills (Fig. 3C). However, when we analysed the male and female data separately, we observed that the male cohort of *Nat9a*^*KO*^ mice spent significantly less time running on rotarod with less distance travelled compared with wild type males (Fig. 3D). This was not seen, however, in the female mice (Fig. 3E) indicating that only the male *Nat9a*^*KO*^ mice have a partial impairment of motor coordination.

### *Nat9a* is expressed in parvalbumin positive interneurons in brain

**Fig. 4:**
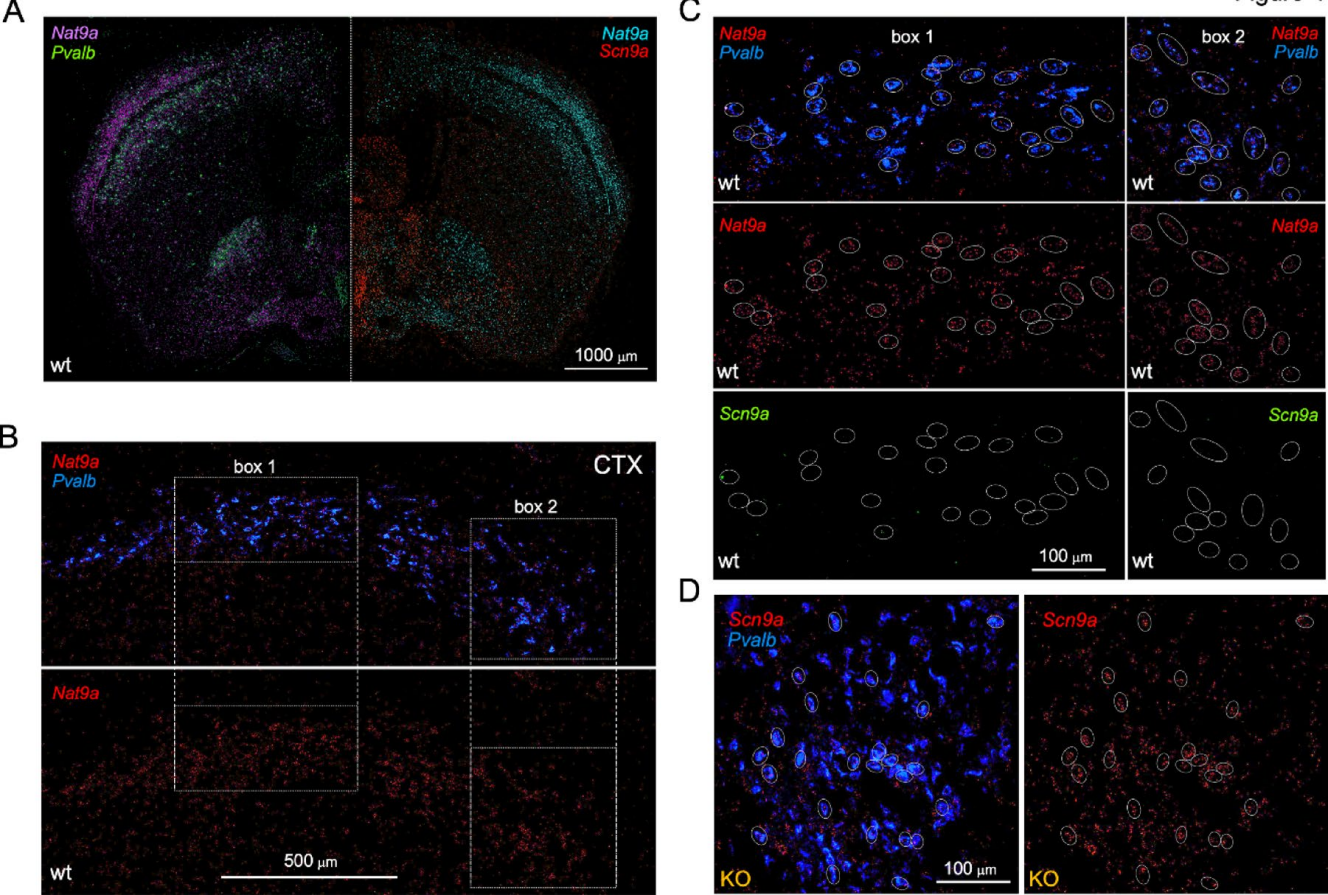
*Nat9a* is expressed in *Pvalb* + inhibitory neurons of cerebral cortex. (**A**) Sections of fresh frozen wild type mouse brain (7–10 μM thick) were analysed by RNAscope assay. Panel A shows localization of *Nat9a* lncRNA (magenta) compared to *Pvalb* mRNA (green) on its left side, and *Nat9a* lncRNA (cyan) compared to *Scn9a* mRNA (red) on its right side. *Nat9a* expression coincides with *Pvalb* + neurons enriched in cortex, amygdala and thalamic reticular nucleus, whereas *Scn9a* mRNA is enriched in the thalamus and hypothalamus, also see Suppl. Figure 6. (B) Localization of *Nat9a* lncRNA (red) compared to *Pvalb* mRNA (blue). *Nat9a* is expressed at a high level in multiple large neurons in layers 2/3 of the cerebral cortex (CTX), many of which (but not all) are *Pvalb* + . (**C**) Enlarged Box1 and Box 2 panels with positions of *Pvalb* + neurons indicated by white circles (wild type mouse). Similar to DRG and spinal cord tissues, data show that *Nat9a* (red) and *Pvalb* (blue) are co-expressed in multiple neurons and *Scn9a* (green) is not expressed in *Pvalb* + cells. (**D**) *Nat9a* knockout leads to a rise in *Scn9a* expression in mouse DRG *Pvalb* + cells. Localization of *Scn9a* mRNA (red) was compared to that of *Pvalb* mRNA (blue). Positions of *Pvalb* + neurons are shown by white circles and demonstrate that *Nat9a* knockout leads to *Scn9a* expression in *Pvalb* + cells.

We next explored if a similar discordant neuronal expression pattern between *Scn9a* and *Nat9a* takes place in brain. RNAscope of wild type and *Nat9a*^*KO*^ mice brain sections showed that indeed there is a distinct discordant pattern of expression with *Scn9a* mRNA expressed primarily in hypothalamic and thalamic neurons, whereas *Nat9a* lncRNA is enriched in neurons of cortex, globus pallidus, amygdala, and thalamic reticular nucleus (Fig. 4A and Supplementary Fig. 7). *Nat9a* lncRNA was strongly expressed in neocortex, especially in the layer 2/3 and layers 5 and 6, the latter layers are in keeping with the typical location of cortical *Pvalb* + interneurons (Fig. 4A and Supplementary Fig. 7).

Similarly, we also observed a discordant expression pattern in brain for *Scn1a* and *Scn9a* mRNAs, with *Scn1a* expression coinciding with that of *Nat9a* in cortex, globus pallidus and thalamic reticular nucleus (Supplementary Fig. 8).

In cortex, all *Pvalb* + interneurons were expressing *Nat9a* lncRNA but none or little *Scn9a* mRNA (Fig. 4B and C) and similar to results described above for DRG and spinal cord tissues there was a clearly detectable rise in *Scn9a* expression in *Pvalb* + cortical neurons in *Nat9a*^*KO*^ mice brain (Fig. 4D).

### *Nat9a* lncRNA is specific to parvalbumin positive type of interneurons

Parvalbumin (Pvalb) expressing GABA-ergic interneurons make up about 40% of all inhibitory neurons, with the other two large groups represented by somatostatin (Sst) and 5HT3aR- expressing neurons which account for ~ 30% of cortical intraneuronal populations each. *Sst* + neurons also populate layers 2/3 and 4 to 6. We next wanted to confirm whether *Nat9a lncRNA* is restricted to *Pvalb* + interneurons as suggested by single-cell genomic data^49^.

**Fig. 5:**
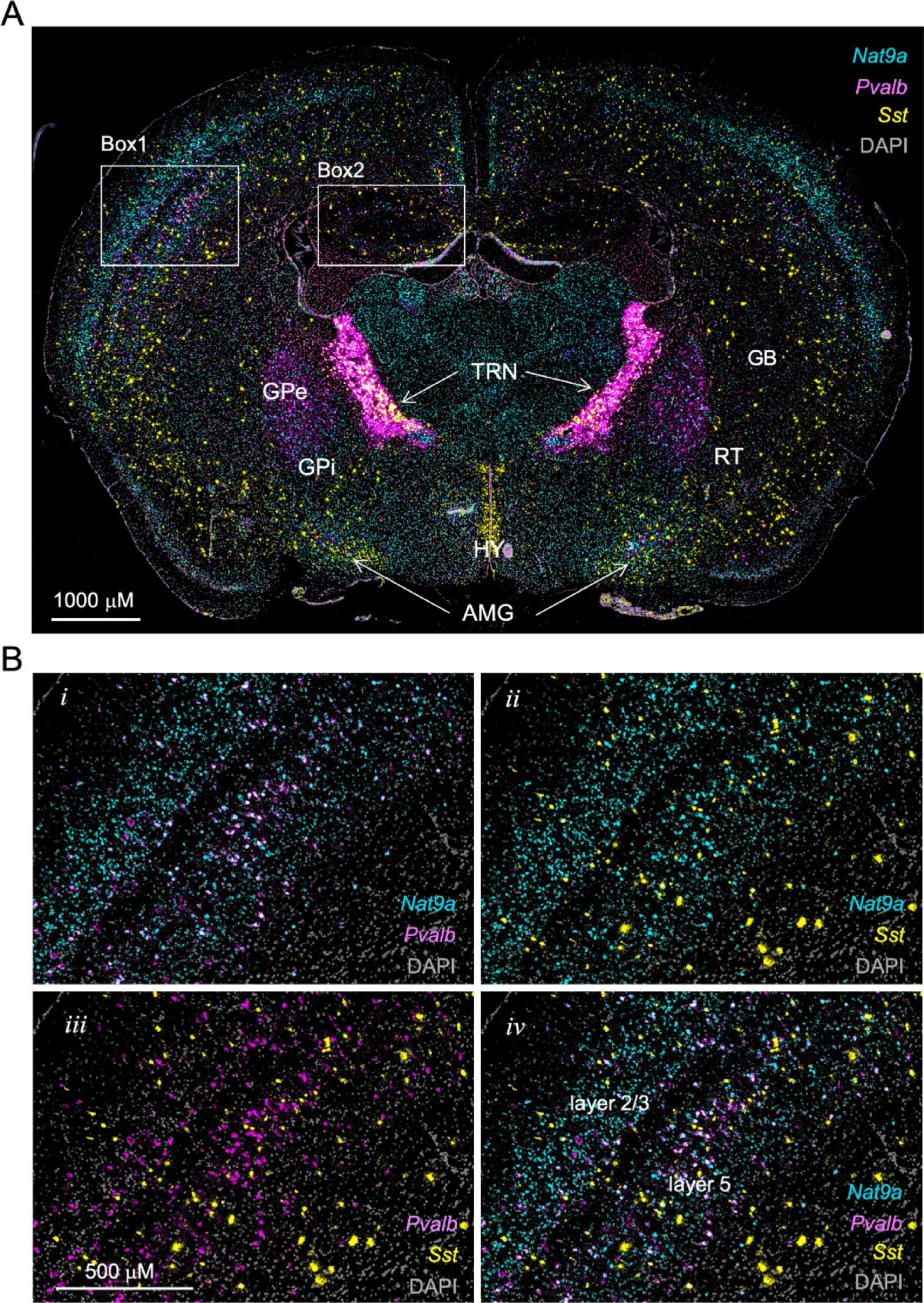
*Nat9a is* expressed in *Pvalb* + but not in *Sst* + inhibitory neurons. (A) Sections of fresh frozen wild type mouse brain (7–10 μM thick) were analysed by RNAscope assay. Panel A shows localization of *Nat9a* lncRNA (cyan) compared to *Pvalb* mRNA (magenta) and *Sst* mRNA (yellow) expression patterns. Both *Pvalb* and *Sst* mRNA are enriched in cortex (Box 1), amygdala (AMG), hippocampus (Box 2, also see Supplementary Fig. 8A), globus pallidus (GP) and especially enriched in reticular nucleus of thalamus (TH). *Nat9a* expression however coincides only with *Pvalb* + neurons but not with Sst + cells, as demonstrated by circling of individual cells in the enlarged panels of Box1 (**Bi-iv**), showing cortex layers 2/3, 4 and 5. TH—thalamus, HY—hypothalamus, CTX—cortex Scale bars are in white.

RNAscope of wild type mice brain sections showed that indeed *Nat9a* expression is co-localised to *Pvalb* + interneurons in cortex, hippocampus, thalamic reticular nucleus, and other compartments of brain, whereas *Sst* + neurons did not show any *Nat9a* expression (Fig. 5A-B and Supplementary Fig. 9A).

**Fig. 6:**
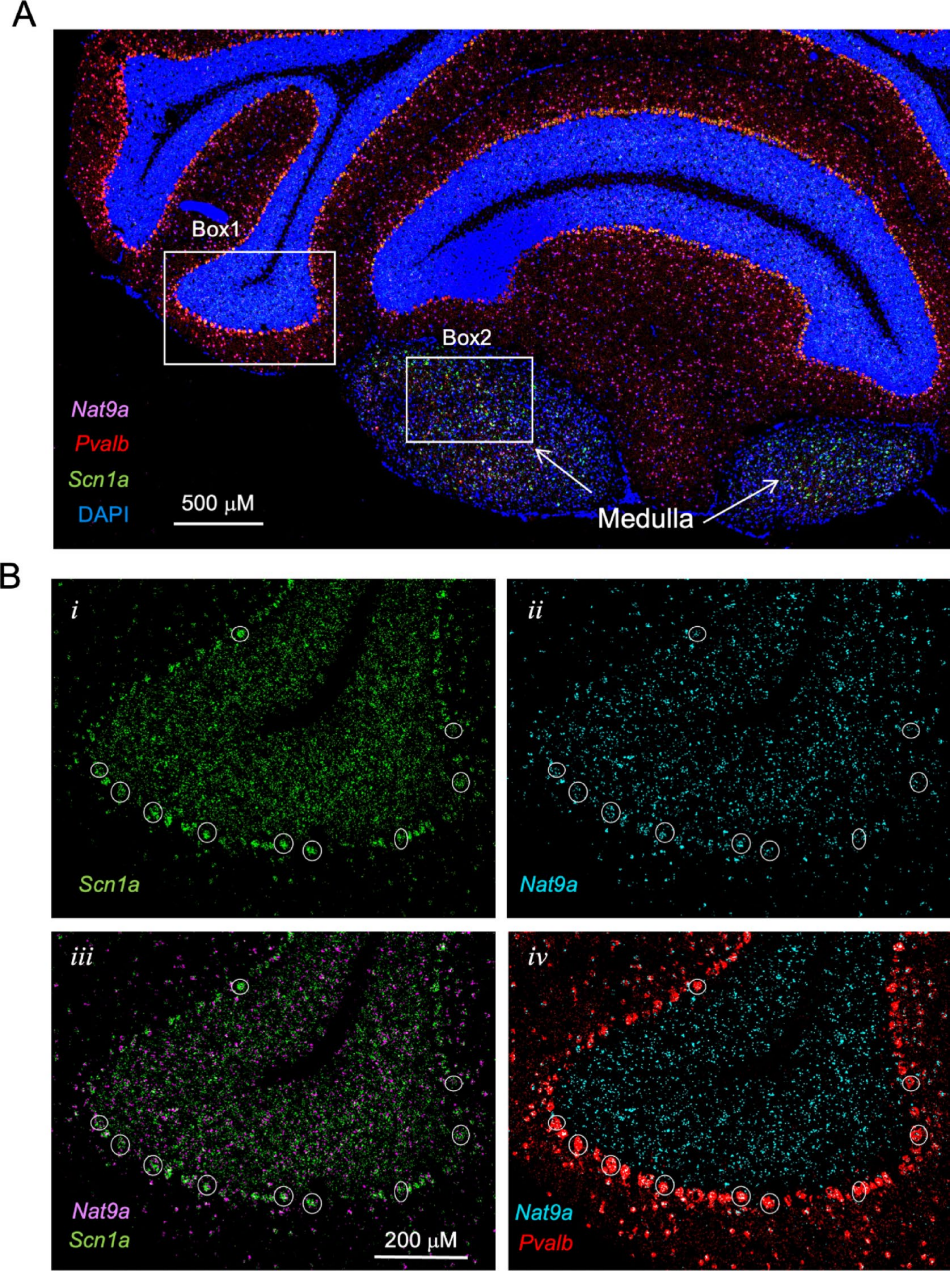
*Nat9a, Scn1a* and *Pvalb* expression patterns in cerebellum and medulla. (**A**) Fresh frozen wild type mouse cerebellum Sects. (7–10 μM thick) were analysed by RNAscope assay. Panel A shows localization of *Nat9a* lncRNA (magenta) compared to *Pvalb* mRNA (red) and *Scn1a* mRNA (green) expression patterns, whilst DAPI staining indicates nuclei positions (blue). All three RNAs are expressed in medulla (Box 2, and in Supplementary Fig. 9B). (**B**) *Nat9a* (cyan) and *Pvalb* mRNA (red) are enriched in large neuronal cells (Purkinje cells) located at the outer edge of cerebellar folium (Box 1, encircled in white, **B,** panels ***ii*** and ***iv***), which is also enriched in *Scn1a* mRNA (green, **B,** panels ***i*** and ***iii***). Scale bars in white.

Similar results for *Nat9a* expression patterns were obtained when analysing cerebellum tissue. *Nat9a* lncRNA expression was enhanced in large *Pvalb* + neuronal cells (Purkinje cells) located at the outer edge of the cerebellar folium (Fig. 6). The *Nat9a* + neurons were also expressing *Scn1a.* There was also a large number of *Nat9a* expressing neurons in medulla areas, most of which were *Scn1a* + and a large number of those were also *Pvalb* + (Fig. 6 and Supplementary Fig. 9B).

### *Nat9a* lncRNA is expressed in *Scn1a* + neurons

**Fig. 7:**
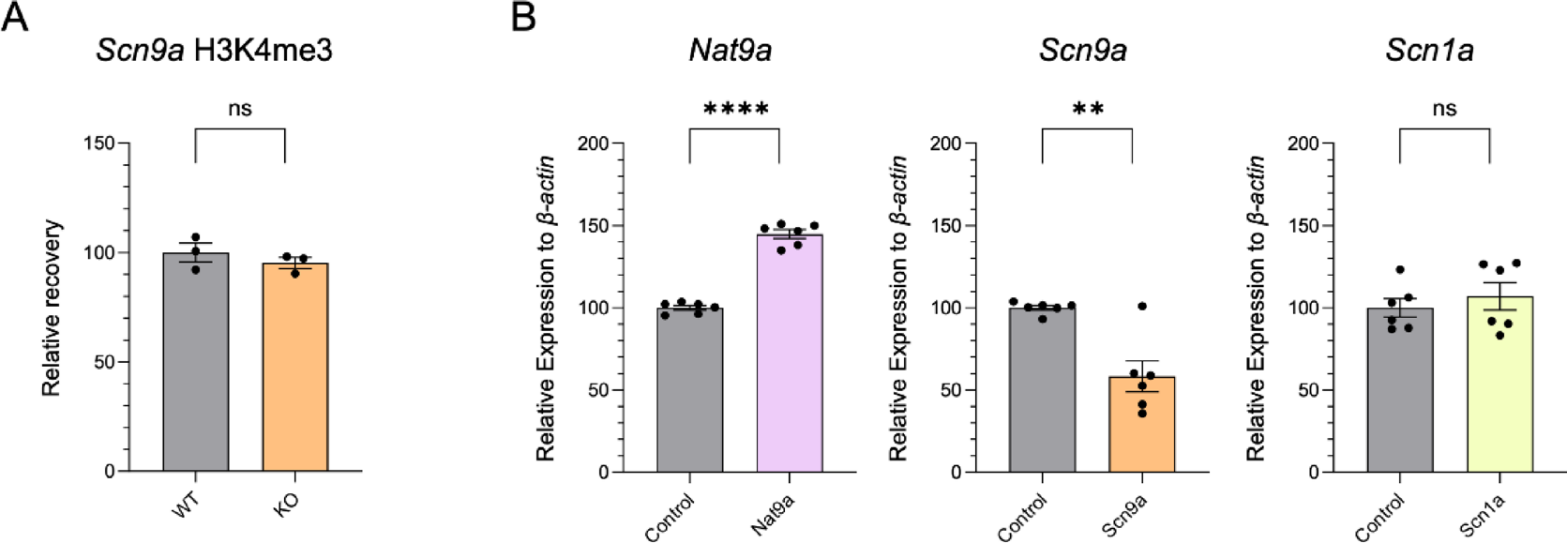
Mechanism of *Nat9a/Scn9a* regulation. (**A**) ChIP-qPCR analysis at the *Scn9a* promoter shows no difference in H3K4me3 post-translational modification between *Nat9a* KO mice and WT littermates in hypothalamic tissue. (**B**) CRISPR activation in Cad cells through targeting of dSaCas9-VPR to the first exon of *Nat9a*, which is distinct from the *Scn1a* promoter region. Driving *Nat9a* expression upwards by ~ 40% (n = 4, **** *p* =  < 0.0001) leads to a downregulation of endogenous *Scn9a* expression by ~ 40% (n = 4, ** *p* =  < 0.01), with no change in *Scn1a* expression. Data-points are denoted by dots, bars show the ± SEM, and data analysed by a Student’s *t*-test.

The expression patterns for *Nat9a* and *Scn1a* in brain and DRG largely overlap and suggest that both genes can be co-regulated (Supplementary Figs. 4B, 8 and 9). Furthermore, the expression patterns of *Scn9a* and *Scn1a*/*Nat9a* are largely discordant. This is supported by single cell RNAseq data from both mouse and human cortex (Supplementary Fig. 10) and mouse DRG (Supplementary Fig. 11C). We observed an upregulation of expression of *Scn9a* in *Nat9a* knockout tissues and used chromatin immunoprecipitation from the hypothalamus to monitor the histone mark, H3K4me3, at the *Scn9a* promoter region. ChIP-qPCR showed no differences between *Nat9a* knockout and wild-type littermates, suggesting that the upregulation in *Scn9a* expression may not be related to chromatin remodelling (Fig. 7A).

Interestingly, when *Nat9a* expression levels were stimulated by CRISPR-induced activation (CRISPRa) using a guide specific to exon 1, this led to a ~ 40% increase in *Nat9a* and a reciprocal ~ 40% decrease in *Scn9a* mRNA levels (Fig. 7B). This would suggest that a straightforward sense/antisense transcriptional competition in the *Nat9a/Scn9a* locus is more likely to provide a basis for the observed discordant expression patterns between *Nat9a* and *Scn9a*, and the divergent bi-directional promoter region between *Scn1a* and *Nat9a* could be the main driver for *Nat9a* lncRNA expression in specific types of neurons, which is supported by a large number of common transcription factors that bind within this region (Supplementary Fig. 11).

Interestingly, there are also a few common transcription factors that bind both *Nat9a* and *Scn9a* promoters (Supplementary Fig. 11), suggesting that once *Nat9a* transcription is shut down the competing opposite direction from *Scn9a* promoter can proceed without any obstacle, something we observe in *Nat9a* KO *Pvalb* + cells.

## Discussion

In this study we explored the functional importance of *Nat9a*, the natural antisense transcript to the *Scn9a* gene using a *Nat9a* global knockout mouse model (*Nat9a*^*KO*^) and provide the first mechanistic insights of *Nat9a* function as a negative regulator of *Scn9a* expression in specific types of neurons. We show that in the peripheral nervous system, DRG *Nat9a* lncRNA (*Gm13629*) expression is limited to *Pvalb* + proprioceptive neurons and is mainly not co-expressed with *Scn9a*. Similarly, *Nat9a* is expressed in *Pvalb* + interneurons in the spinal cord and brain. Neuronal expression of *Nat9a* and *Scn9a* is discordant, that is cells expressing *Nat9a* lncRNA tend to have little or no *Scn9a* mRNA and vice versa in both the PNS and CNS. This expression pattern appears to be largely conserved between mouse and human as per data available from single cell RNAseq for DRG^49,50^, hypothalamus^51^ and in cortex in the Allen Brain Map (Supplementary Fig. 10)^52^. However, there are cases such as in the human cortex where *SCN9A* and *NAT9A* (*LOC101929680*) appear to be co-expressed (Supplementary Fig. 10). Further work is needed to understand whether the same molecular mechanism observed in mouse is present in humans. However, it is important to highlight that the first exon of the human *NAT9A* gene is within an intronic region of *SCN1A*^14^ instead of being adjacent to *Scn1a* exon 1 in the mouse genome and so the human mechanism could be more complex.

We demonstrated that elimination of *Nat9a* expression in the *Nat9a KO* led to a rise in *Scn9a* expression in *Pvalb* + neurons in both the PNS and CNS, indicating that *Nat9a* transcriptional flow and/or the *Nat9a* lncRNA negatively regulates *Scn9a* transcription. This regulation is not achieved by changes in *Scn9a* histone H3K4me3 modification indicating that a direct transcriptional interference between *Nat9a* and *Scn9a* genes is the most likely underlying mechanism. Deletion of the *Nat9a* gene did not affect the pain sensitivity of mice lacking *Nat9a*, in keeping with the fact that *Nat9a* lncRNA is not expressed in classically nociceptive sensory neurons but in proprioceptive *Pvalb* + neurons, as previously published in single cell RNAseq data for mouse DRG^49,50,53^.

*Nat9a* knockout, however, resulted in partially impaired motor coordination in male mice suggesting that *Nat9a* levels are important for the proper function of *Pvalb* + interneurons and proprioceptors. Notably, *Nat9a* expression in *Pvalb* + neurons coincides with that of the *Scn1a* gene (Na_V_1.1 sodium channel) with both genes sharing a divergent bi-part promoter region. This suggests a mechanism by which *Nat9a* transcription leads to suppression of *Scn9a* and promotes neuronal expression of Na_V_1.1 over Na_V_1.7.

### *Nat9a* transcription suppresses and competes with *Scn9a*

A common scenario for a negative regulatory mechanism by an antisense RNA at the transcriptional level may involve direct transcriptional interference, where (*i*) active transcription complexes of both strands physically interfere with each other either via transcriptional collision and/or (*ii*) competition for transcription factors between sense and antisense strands’ promoters^16,17,28,54–57^. Transcriptional collision occurs when both sense and antisense genes are actively transcribed at the same time and RNA polymerase complexes on convergent directions act as a physical barrier to halt the action of each other, thus resulting in termination of the transcription^54–57^. The level of transcription initiation would also depend on transcription factors loading onto competing promoters^28,54^. Finally, the other mechanistic possibility of the antisense lncRNA-dependent negative transcriptional regulation is local chromatin remodelling, in which the antisense transcript may recruit DNA and histone methyltransferases to the sense gene’ promoter region, like in a case described for the Kv7.1 potassium channel gene regulation by the *Kcnqot1* antisense transcript^58–60^.

Our data clearly showed that *Nat9a* and *Scn9a* expression is largely discordant, however there was no changes in the chromatin histone H3K4me3 mark at the *Scn9a* promoter in analysed *Nat9a* KO mouse hypothalamic tissue (Fig. 7A). The data indicates that whilst expression of *Nat9a* suppresses *Scn9a* transcription the most likely mechanistic scenario is the transcriptional interference (competition) and that in cells such as Pvalb + neurons the *Nat9a* promoter’ efficiency outcompetes the one for *Scn9a*, whilst in nociceptive sensory neurons the situation is reversed. This is also supported by the fact that negating *Nat9a* transcription by knockout leads to a rise of *Scn9a* expression in cells where it was normally suppressed by *Nat9a.* Furthermore, CRISPR-induced activation of *Nat9a* transcription by dSaCas9-VPR leads to an ~ 40% decrease in *Scn9a* expression levels in cells where *Scn9a* transcription typically outperforms that for *Nat9a* (Fig. 7B), suggesting again that a competition in respective promoters’ efficiency allows different cells to shift the balance either towards *Scn9a* in nociceptive sensory neurons or towards *Nat9a* in proprioceptors and interneurons. Interestingly, *Nat9a/Scn1a* and Scn*9a* promoters share some common transcription factors (TFs) which could explain why *Scn9a* levels can rise once the competing direction of *Nat9a* is negated (Supplementary Fig. 11).

However, in proprioceptors and Pvalb + interneurons, *Nat9a* promoter efficiency is likely to be amplified by a larger availability of specific TFs and fully active transcription of *Scn1a*, thus allowing the *Nat9a/Scn1a* divergent promoter to outperform competition from the *Scn9a* promoter (Supplementary Fig. 10).

At this stage we can envisage that the choice between expression of *Scn9a* and *Scn1a/Nat9a* genes could be made during the development process and later after differentiation through specific TFs. The bubble plot (Supplementary Fig. 11C) shows that there are two recognisably distinct populations of TFs that are either expressed in *Scn1a* + and *Pvalb* + DRG neurons, or those TFs that are primarily expressed in *Scn9a* + DRG neurons.

This, at least in part, may explain the genetic background of making the choice between *Nat9a/Scn1a* vs *Scn9a* expression*.* We also cannot exclude that even though *Nat9a/Scn1a* and *Scn9a* promoters’ sequences show the presence of motifs for the same TFs (Supplementary Fig. 11B), these TFs may have an opposite effect on those promoters, e.g., activate *Nat9a/Scn1a* but repress *Scn9a,* or vice versa.

We still cannot rule out a possibility for *Nat9a* lncRNA influencing the efficiency of *Scn9a* mRNA translation which has been observed for some antisense transcripts^61–64^. This however has typically been achieved via 5′-end overlap, whilst *Nat9a* and *Scn9a* have complementary homology at their respective 3′-ends. Similarly, a possibility for *Nat9a* to modulate *Scn9a* splicing via RNA masking which is shown for some sense-antisense pairs is also unlikely, as our data show a paucity of *Scn9a* mRNA in neurons expressing *Nat9a*^17^.

### Nat9a is expressed in specific types of neurons

Our data show that *Nat9a* is expressed in Pvalb + proprioceptors in the PNS and inhibitory type Pvalb + neurons in the CNS. In both cases, *Nat9a*-expressing *Pvalb* + neurons have very low/undetectable expression of *Scn9a*. Thus, the PSNF2 type neurons in mice (proprioceptors, *Pvalb* + DRG neurons) and the equivalent human DRG cells show low or undetectable *Scn9a*/*SCN9A* expression^49,53,65^ (Fig. 2 and Supplementary Fig. 4). This is in keeping with the role of proprioceptors as non-nociceptive low-threshold mechanoreceptors in DRG and that Nav1.7 inhibitors or loss of function mutations in CIP patients do not affect proprioception^10,66^.

Our RNAscope data and accompanying analysis of single cell RNAseq data confirm that across the DRG, *Scn9a* is predominantly expressed in non-*Pvalb* + neurons although a subset of *Pvalb* + neurons do still express *Scn9a*, albeit at a lower level (Supplementary Fig. 5A-E). In contrast, Espino et al. report that *Scn9a* is ubiquitously expressed in proprioceptors^67^. Possible reasons for the incongruity of the two studies could be related to differences in RNAscope methodologies and/or the use of a *Pvalb*-Cre reporter line that designates the *Pvalb* + lineage cells, making a direct comparison of the two studies difficult.

In brain, *Nat9a* is expressed highly in hypothalamus, thalamus, and cortex (Figs. 4 and 5) and the distribution of *Nat9a*-expressing neurons in cortex is enriched in layer 2/3 and layers 5 and 6, in keeping with the typical locations of the Pvalb + basket and chandelier type GABA-ergic interneurons in this area (Figs. 4, 5 and Supplementary Figs. 7 and 8)^68^. Similarly, *Nat9a* expression is co-localised with *Pvalb* + interneurons in cerebellum (Fig. 6).

In all cases *Nat9a* can typically be co-localized to cells expressing *Scn1a*, but not to interneurons expressing *Sst*, suggesting that the *Nat9a* role in suppressing *Scn9a* levels is specific to Pvalb + type interneurons. There are still large numbers of cells in spinal cord and brain that express high levels of *Nat9a* but not *Pvalb*, suggesting that *Nat9a*-based suppression of *Scn9a* expression is employed by other types of cells too. Those cells are shown to express *Scn1a*, indicating that the *Nat9a* promoter is active in any *Scn1a* + cells most likely due to the divergent bi-part promoter for *Scn1a* and *Nat9a*.

The discordant expression between *Nat9a/Scn1a/Pvalb* and *Scn9a* appears to be largely conserved between mouse and human in the cortex, thus *Scn9a* levels are down in Pvalb + neurons whilst expression for both *Nat9a* and *Scn1a* is high (Supplementary Fig. 10)^49,53,65^. Finally, data from hypothalamus show a similar picture in which hypothalamic Pvalb + interneurons show high *Nat9a* and low or no *Scn9a* expression^51^.

### *Nat9a* is expressed in specific cells to prevent potential Na_V_1.7 interference with Na_V_1.1?

*Nat9a* lncRNA expression is driven from a bidirectional promoter region shared with *Scn1a,* which encodes the Na_V_1.1 sodium channel that is essential for normal proprioceptive signalling and motor behaviours^67,69^. Na_V_1.1 also plays a predominant role in excitability of fast-spiking *Pvalb* + interneurons and is needed for action potential (AP) initiation, propagation and setting the threshold for APs^70,71^. Our results suggest that in fast spiking GABA-ergic *Pvalb* + interneurons (CNS) and *Pvalb* + proprioceptors (PNS) there is a transcription regulation mechanism that suppresses *Scn9a* and therefore prevents (or severely limits) production of Na_V_1.7 by initiating natural antisense *Nat9a* transcription. This most likely eliminates potential interference by Na_V_1.7 into the functional role of Na_V_1.1 in proprioceptors and interneurons. In keeping with this, we observed a significant rise in *Scn9a* expression levels in *Nat9a*^*KO*^ mice in cells and tissues normally expressing *Nat9a*.

The deletion of *Nat9a* and rise in *Scn9a* expression in proprioceptors and Pvalb + interneurons did not affect the pain sensitivity of mice lacking *Nat9a* but led to a partially impaired motor coordination in male *Nat9a*^*KO*^ mice. Why this affected male but not female knockouts is unclear but could be related to hormonal regulation of voltage-gated sodium channels (e.g. estradial has been shown to upregulate Na_V_1.7 in sensory neurons)^72^. The effects of different sex hormones in the *Nat9a*^*KO*^ mice requires further investigation but it is tentative to suggest that the rotarod results in the males was due to interference between Na_V_1.7 and Na_V_1.1 in proprioceptors and/or in Pvalb + interneurons. Interneurons in spinal cord were previously reported to be having an important role in motor coordination, albeit the effect of their negation is subtle and was only detected clearly when animals were tested whilst swimming, thus engaging their limbs and tails in a fast and coordinated manner^73,74^.

The selective expression of Na_V_1.1 over Na_V_1.7 in Pvalb + interneurons could be explained by differences in the electrophysiological properties of these channels. Whilst both Na_V_1.7 and Na_V_1.1 exhibit rapid activation and deactivation kinetics, Na_V_1.7 recovers from inactivation with a substantially slower time constant than Na_V_1.1^75–77^. This slower recovery makes Na_V_1.7 more suitable for nociceptive signalling in sensory neurons rather than for the rapid, repetitive firing required in interneurons, for which Na_V_1.1 is better suited. Therefore, the ectopic presence of Na_V_1.7 in Pvalb + interneurons and other cells that rely on fast recovery of sodium channels could impair their function. Indeed, mutations in *SCN1A* that prolong recovery from inactivation and reduced action potential firing lead to impaired sodium channel activity in interneurons^78^.

The experiments described here were primarily designed to see whether *Nat9a*^*KO*^ mice are deficient in nociception, yet future experiments on cognitive behaviour, more sophisticated motor-coordination tests and single cell electrophysiology of neurons expressing both Na_V_1.1 and Na_V_1.7 could uncover in detail the consequences of Na_V_1.7 interference with Na_V_1.1 function in those neurons.

Our work focussed on characterising the mouse *Nat9a* gene. The comparable gene in humans is represented by transcript NR_110260 (*SCN1A-AS*), which overlaps both the *SCN9A* and *SCN1A* genes. Similar to what we observe in mouse, there is a positive correlation in expression of *SCN1A* and *SCN1A-AS* in human pediatric brain samples taken from patients with drug-resistant epilepsy^79^. It is interesting to note that some human epilepsy disorders have been linked to variants in *SCN9A*, although whether they directly contribute to the phenotype remains controversial^80^. It will interesting to explore whether any of these *SCN9A* variants affect *NAT9A*/*SCN1A* transcription. Analogously, in a DRG conditional *Scn9a* KO we showed that *Scn1a* is upregulated as a compensatory mechanism^13^.

In summary, we show that *Nat9a* lncRNA expression suppresses transcription of *Scn9a* (Na_V_1.7) in cells where *Scn1a* (Na_V_1.1) is the main sodium channel so that Na_V_1.7 does not interfere with Na_V_1.1 function. Thus transcription of *Nat9a* lncRNA allows for precise cell-specific regulation of *Scn9a* expression and provides a mechanism to suppress ectopic expression of Na_V_1.7 in *Pvalb* + /*Scn1a* + neurons.

## Supplementary Information

Below is the link to the electronic supplementary material.

Supplementary Material 1

Supplementary Material 2

Supplementary Material 3

## Data Availability

The sequences (accession numbers KM096552, KM096553) and datasets analysed during the current study are available in GenBank (https://www.ncbi.nlm.nih.gov/genbank/), JASPAR (https://jaspar.elixir.no/), the Harmonized DRG and TG reference atlas (https://painseq.shinyapps.io/harmonized_painseq_v1/) and the Allen Brain Map single cell RNAseq data repository (https://brain-map.org/our-research/cell-types-taxonomies/cell-types-database-rna-seq-data).
