# Supplementary material for: Natural antisense transcript Nat9a suppresses Scn9a (NaV1.7) expression in parvalbumin-positive proprioceptive and inhibitory neurons
Source: Sci Rep. 2026 Apr 16;16:17733. doi: 10.1038/s41598-026-48500-8 (PMC13247247; doi:10.1038/s41598-026-48500-8)
Supplement: Supplementary file 2 — Supplementary Material 2 [file 41598_2026_48500_MOESM2_ESM.pdf]

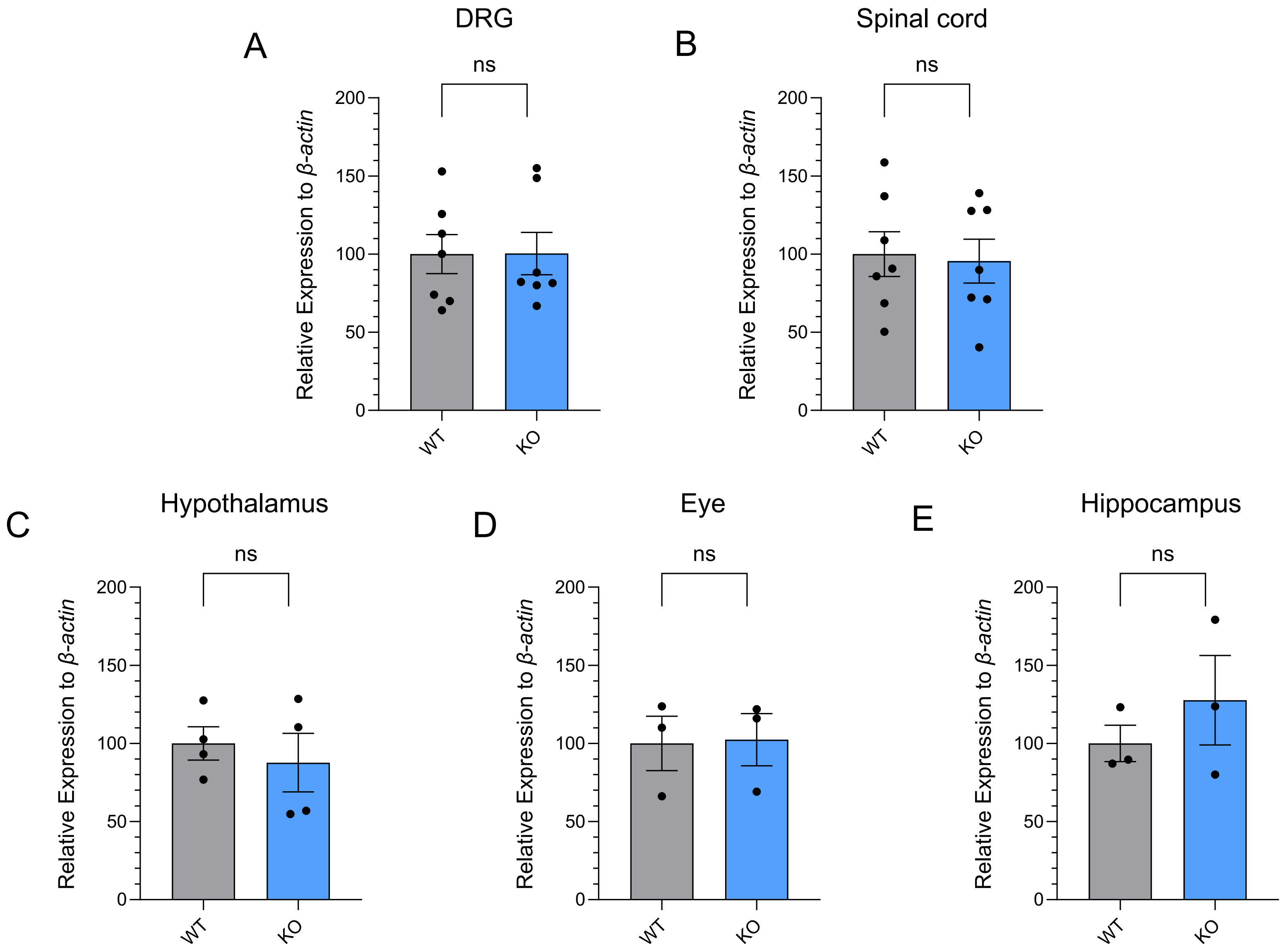

Suppl . Fig S1. ***Scn1a*** **expression is not affected in the** ***Nat9a***^{KO} **mouse line**
Scn1a mRNA level in *Nat9a* knockout and wild type littermate controls determined by qPCR in (**A**) DRG (n=7); (**B**) Spinal cord (n=7); (**C**) Hypothalamus (n=4); (**D**) Eye (n=3) and (**E**) Hippocampus (n=3).

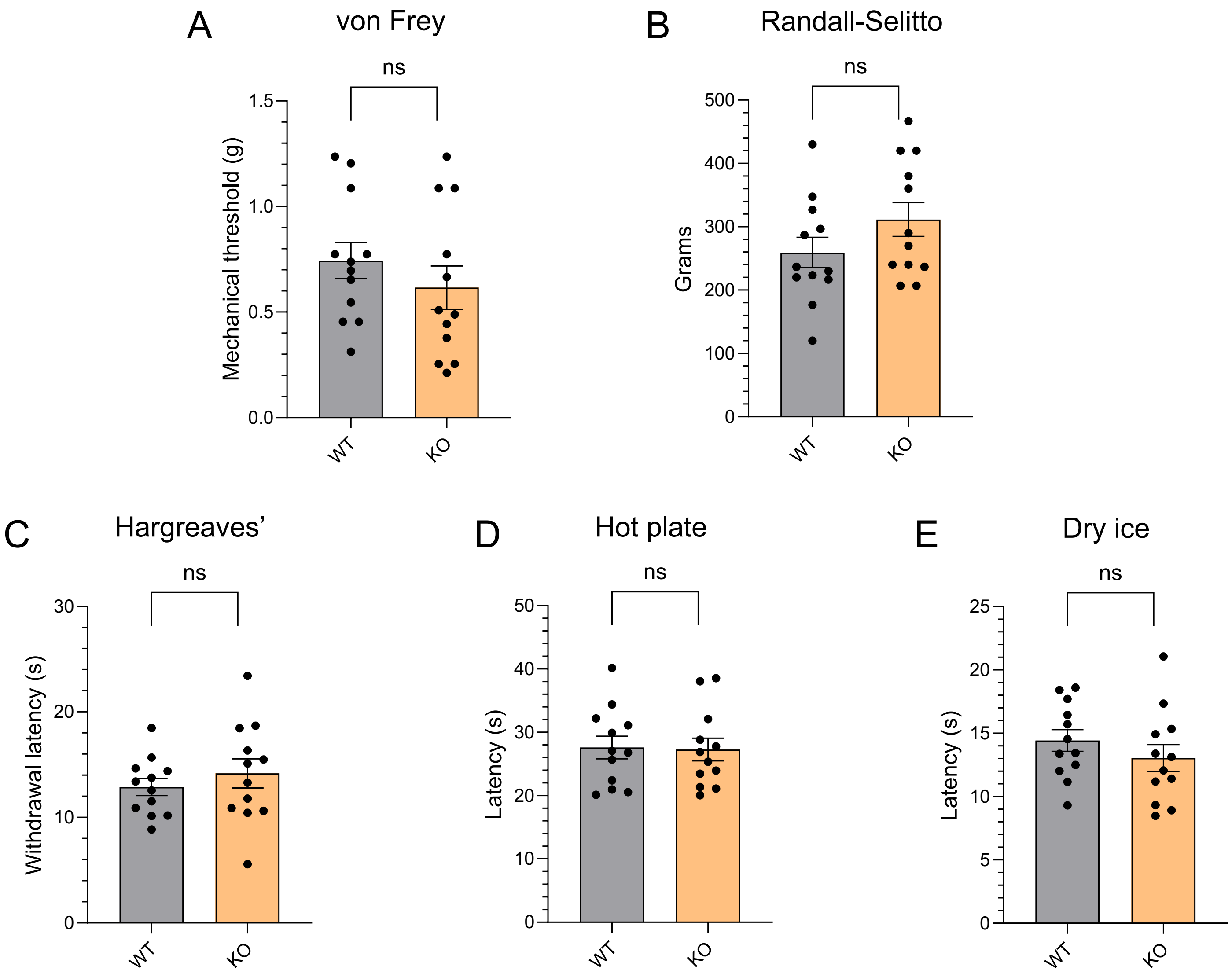

Suppl. Fig S2A. ***Nat9a^{KO}*** **mice show normal acute pain behaviour (mixed sexes)**

(**A**) Mechanical withdrawal threshold to von Frey filaments; (**B**) response to noxious mechanical stimuli evoked by Randall Selitto apparatus on the tail; (**C**) withdrawal latency to noxious heat in the Hargreaves' test; (**D**) response to hot plate at 50°C; and (**E**) cold plantar assay in *Nat9a* knockout mice (n=12; male KO=8, female KO=4) compared to the wild type littermate controls (n=12; male WT=8, female WT=4).

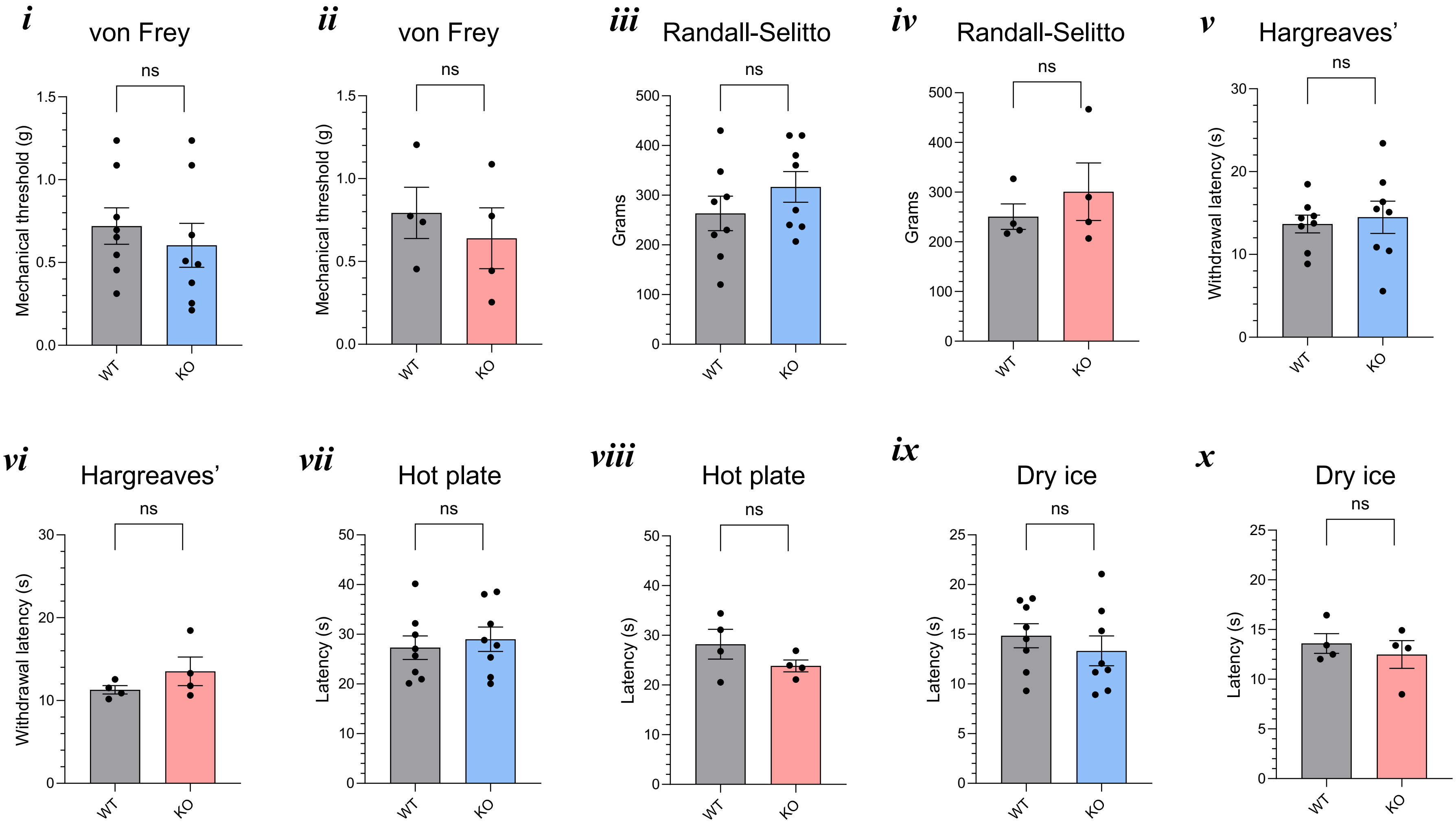

Suppl. Fig S2B *Nat9a*^{KO} mice show normal acute pain behaviour (males and females separated)
Mechanical (***i-iv***) and thermal responses (***v-x***) in male (blue column, n=8) and female (pink column, n=4) *Nat9a* knockout mice compared to the respective male wild type littermate controls (grey column, n=8) and female (grey column, n=4). Separate tests results are shown for withdrawal threshold to von Frey filaments (***i-ii***); response to noxious mechanical stimuli evoked by Randall-Selitto apparatus on the tail (***iii-iv***); withdrawal latency to noxious heat in the Hargreaves' test (***v-vi***); response to hot plate at 50°C (***vii-viii***); and the cold plantar assay (***ix-x***).

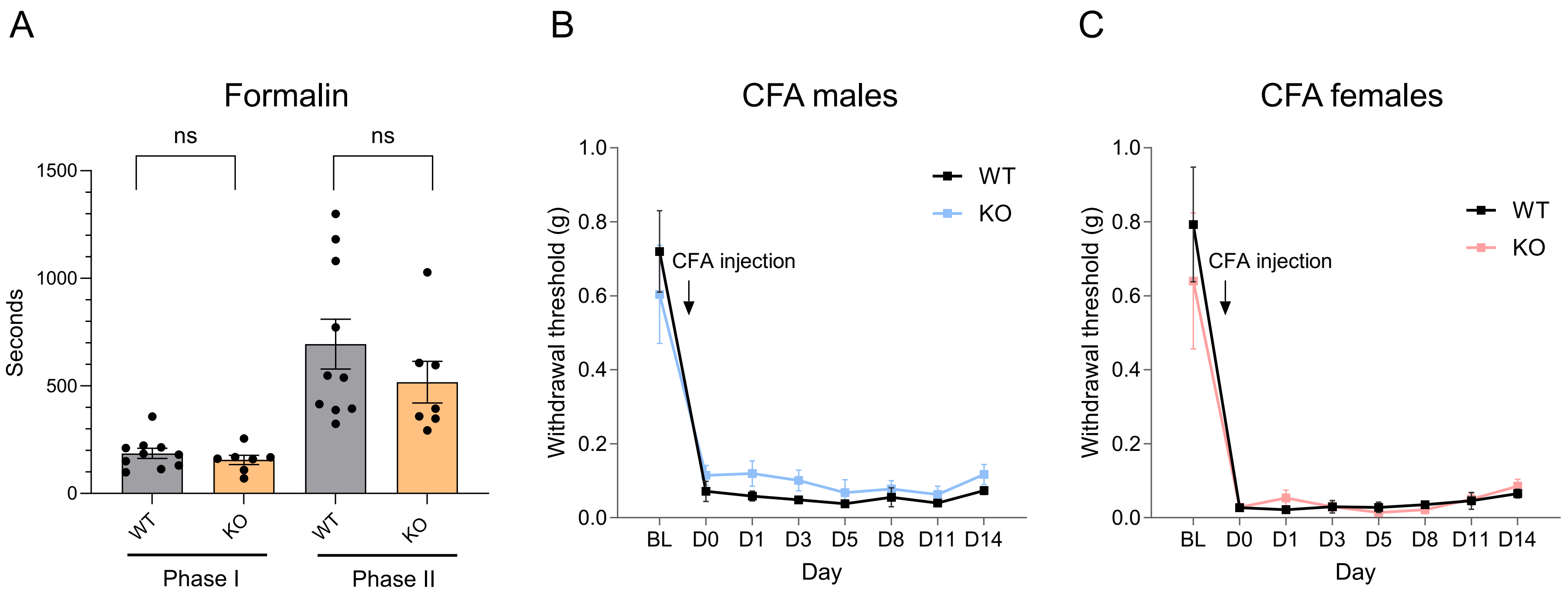

Suppl. Fig S3 ***Nat9a^{KO}* mice show no changes in inflammatory pain behaviour.**
(A) Behavioural responses of *Nat9a* knockout (n=7, male=1, female=6) and wild type littermate (n=10, male=5, female=5) after intraplantar injection of 5% formalin (20µl). Time spent displaying pain behaviour was measured in seconds over 0-10 mins (phase I) and 10-60 mins (phase II) post-injection. (**B, C**) Withdrawal threshold measured using von Frey after 20µl intraplantar injection at 4 hrs (D0), day 1, day 3, day 5, day 8, day 11 and day 14 post-intraplantar injection of 20µl CFA (B: n=8 male WT, 8 male KO; C: n=4 female WT and 4 female KO). BL=baseline measurement prior to CFA injection.

A

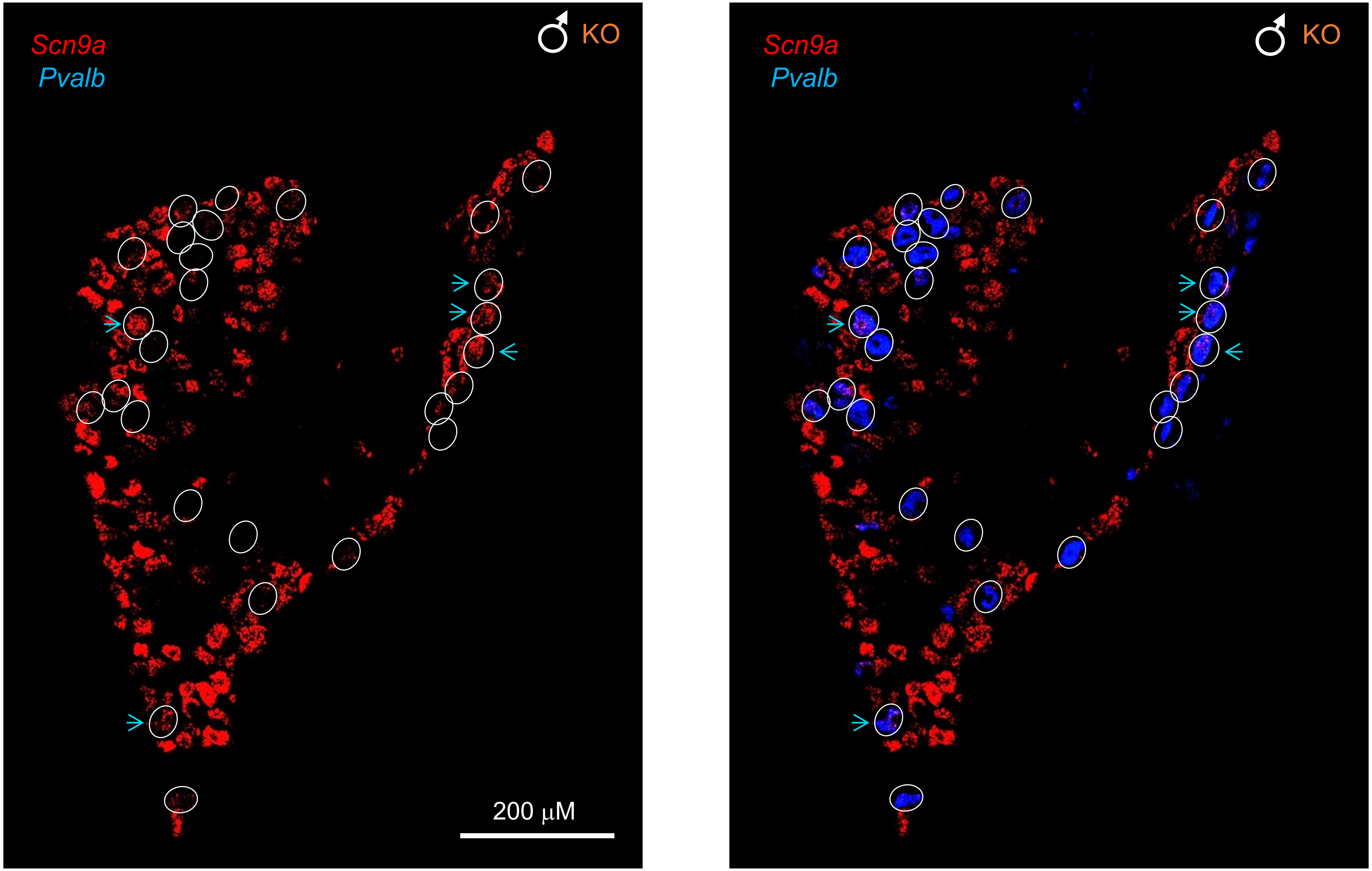

Suppl. Fig S4A: ***Nat9a* knockout leads to a rise in *Scn9a* expression in mouse (male) DRG *Pvalb*+ cells.** Cuts of fresh frozen *Nat9a*^{KO} mouse (male) DRG node sections (~10 µM thick) were analysed by RNAscope assay. Localization of *Scn9a* mRNA (red) was compared to *Pvalb* mRNA (blue) localization. Positions of *Pvalb*+ neurons are shown by white circles and demonstrate that *Nat9a* knockout leads to *Scn9a* expression in *Pvalb*+ cells (marked by cyan arrows). Scale bar is in white.

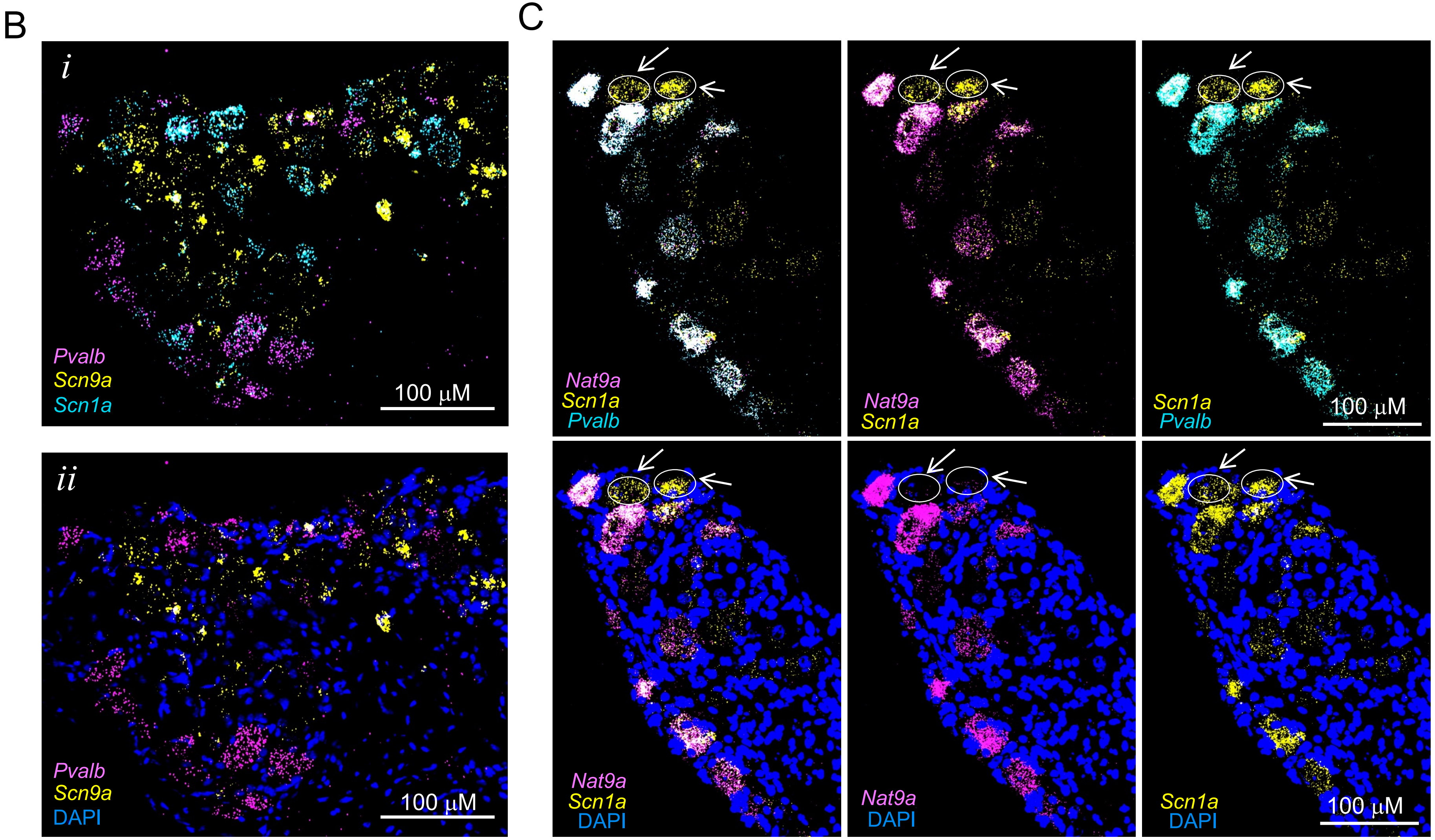

Suppl. Fig S4B and C: ***Scn9a, Scn1a*** **and** ***Nat9a*** **expression patterns in mouse DRG.** (**B**) *Scn9a* and *Scn1a* show discordant expression patterns as shown by RNAscope assay. Localization of *Scn9a* mRNA (yellow) was compared to *Scn1a* mRNA (cyan, panel **B*i***) and *Pvalb* mRNA (magenta, panel **B*ii***) localization. *Scn1a* expressing neurons are distinct from those expressing *Scn9a*. *Pvalb+* neurons (proprioceptors) express *Scn1a* but not *Scn9a*. (**C**) Most of the *Scn1a* expressing neurons (in yellow) also express *Nat9a* (in purple) and *Pvalb* (in cyan), apart from a few cells that express *Scn1a* only (indicated with white circles and arrows), suggesting that the *Scn1a* divergent promoter does not necessarily drive *Nat9a* expression in all cells where it is active. Scale bars are in white.

D

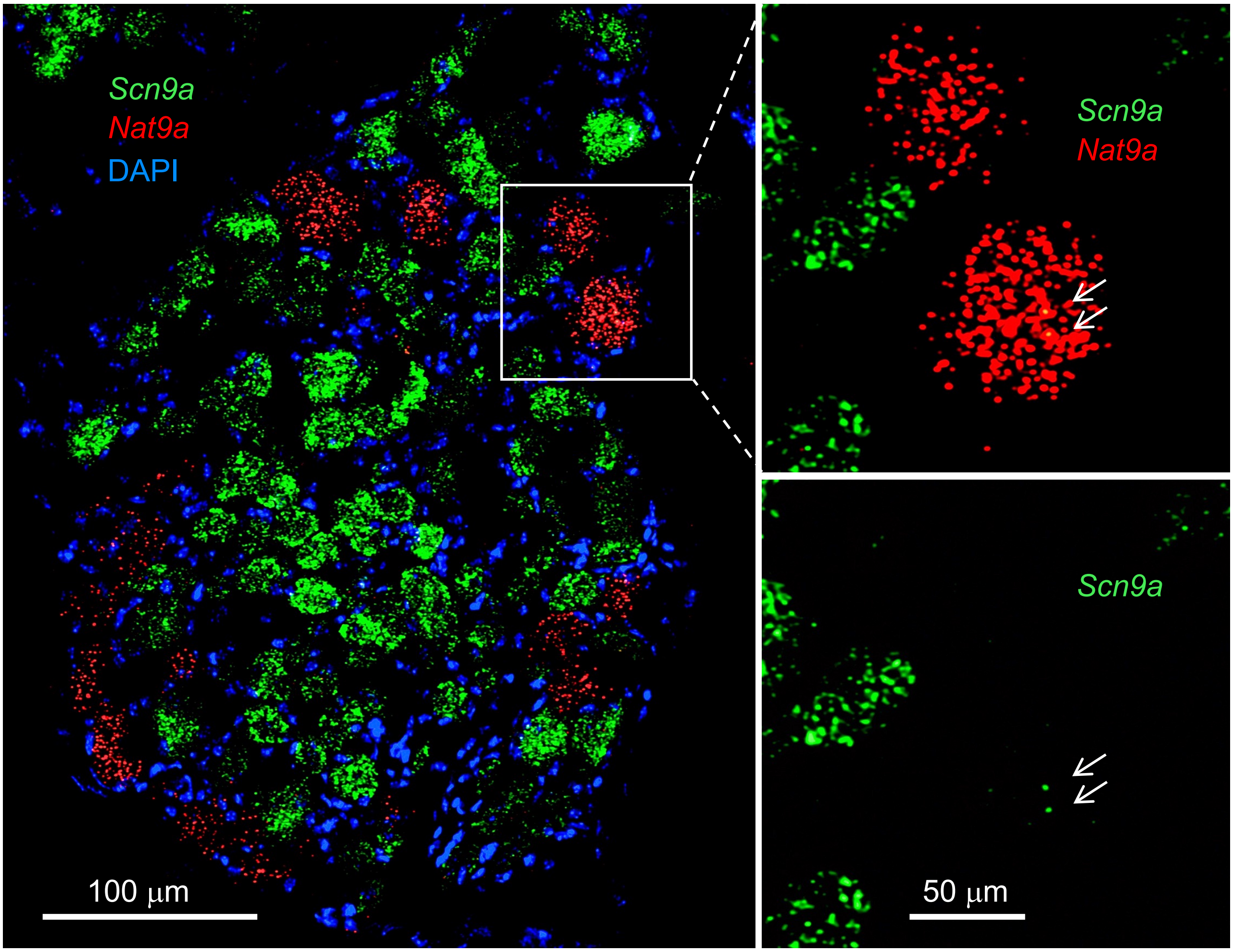

Suppl. Fig 4D: ***Nat9a positive neurons show low level of Scn9a* expression.** Mouse DRG sections (~10 μM thick) were analysed by RNAscope assay. Cells showing high levels of *Nat9a* lncRNA (red, Opal570) were checked for the presence of *Scn9a* mRNA (green, AF488). Some *Nat9a+* cells show low level of *Scn9a* mRNA, as demonstrated on right side panels. *Scn9a* mRNA signal is indicated with white arrows. Positions of nuclei are shown by DAPI staining (blue). Scale bars are in white.

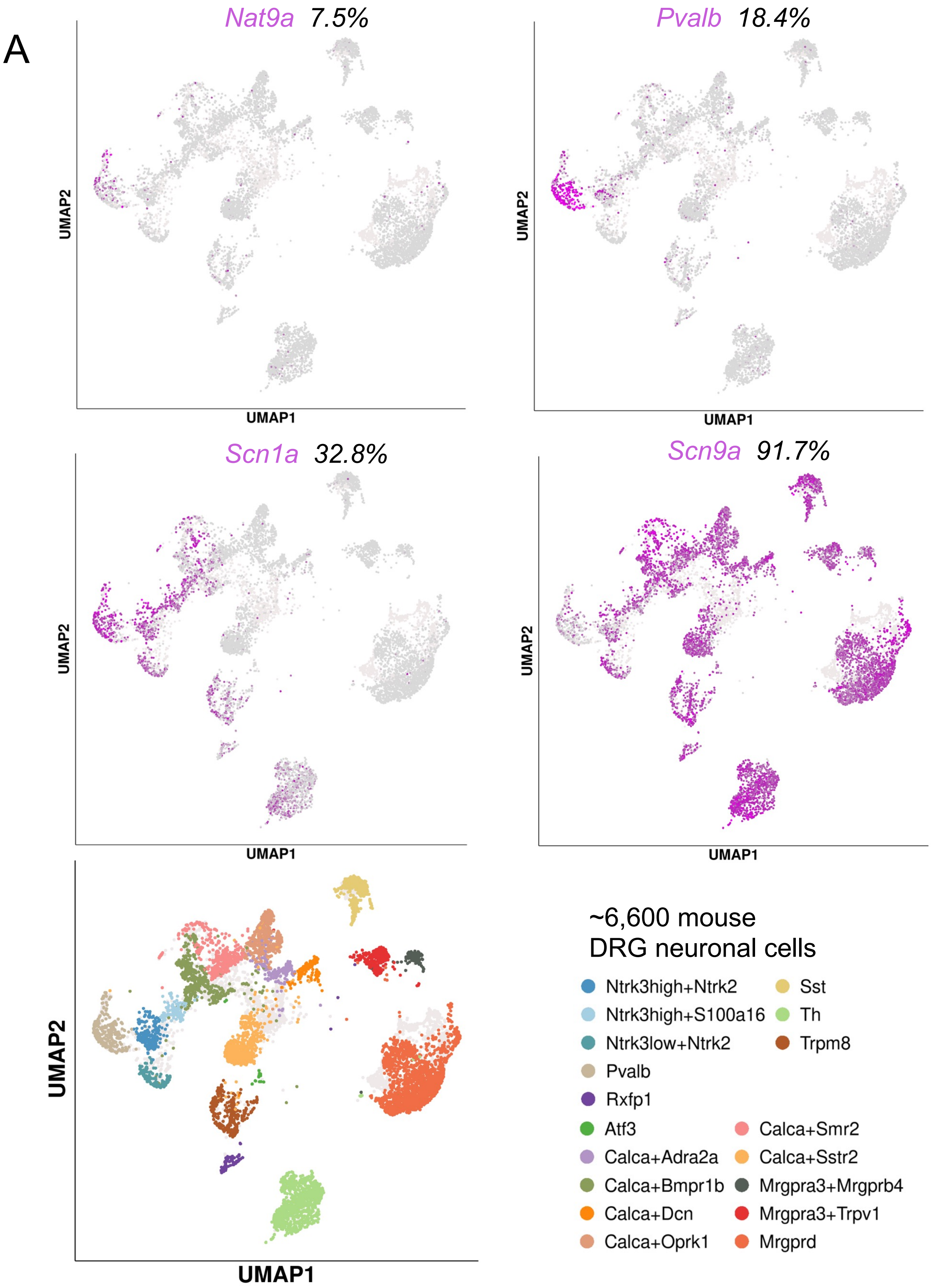

Suppl. Fig 5 A, B: **Single cell transcriptomics analysis.** (**A**) Harmonized mouse DRG RNAseq data [50] were used to assess *Nat9a*, *Scn1a*, *Scn9a* and *Pvalb* RNA expression in subpopulations of DRG neurons. The data (from ~6,600 cells) show that *Nat9a* lncRNA is expressed in ~7.5% of neurons, *Scn1a* mRNA – in 32.8%, *Scn9a* mRNA – in 91.7%, and 18.4% of neurons are *Pvalb*+. (**B**) Co-expression of *Nat9a*, *Scn1a*, *Scn9a* and *Pvalb* RNAs in DRG neurons show that about a half of *Nat9a*+ neurons are also *Pvalb*+, with majority of *Nat9a*+ neurons are in *Pvalb*+ proprioceptors population. Similar numbers of *Nat9a*+ neurons also show some *Scn9a* expression, albeit their subpopulation patterns are different. Almost 2/3-ds of *Nat9a*+ neurons overlap with those expressing *Scn1a* in *Pvalb*+ proprioceptors. Data visualisation was made possible by Harmonized DRG and TG Reference Atlas [50].

Suppl. Fig 5 B

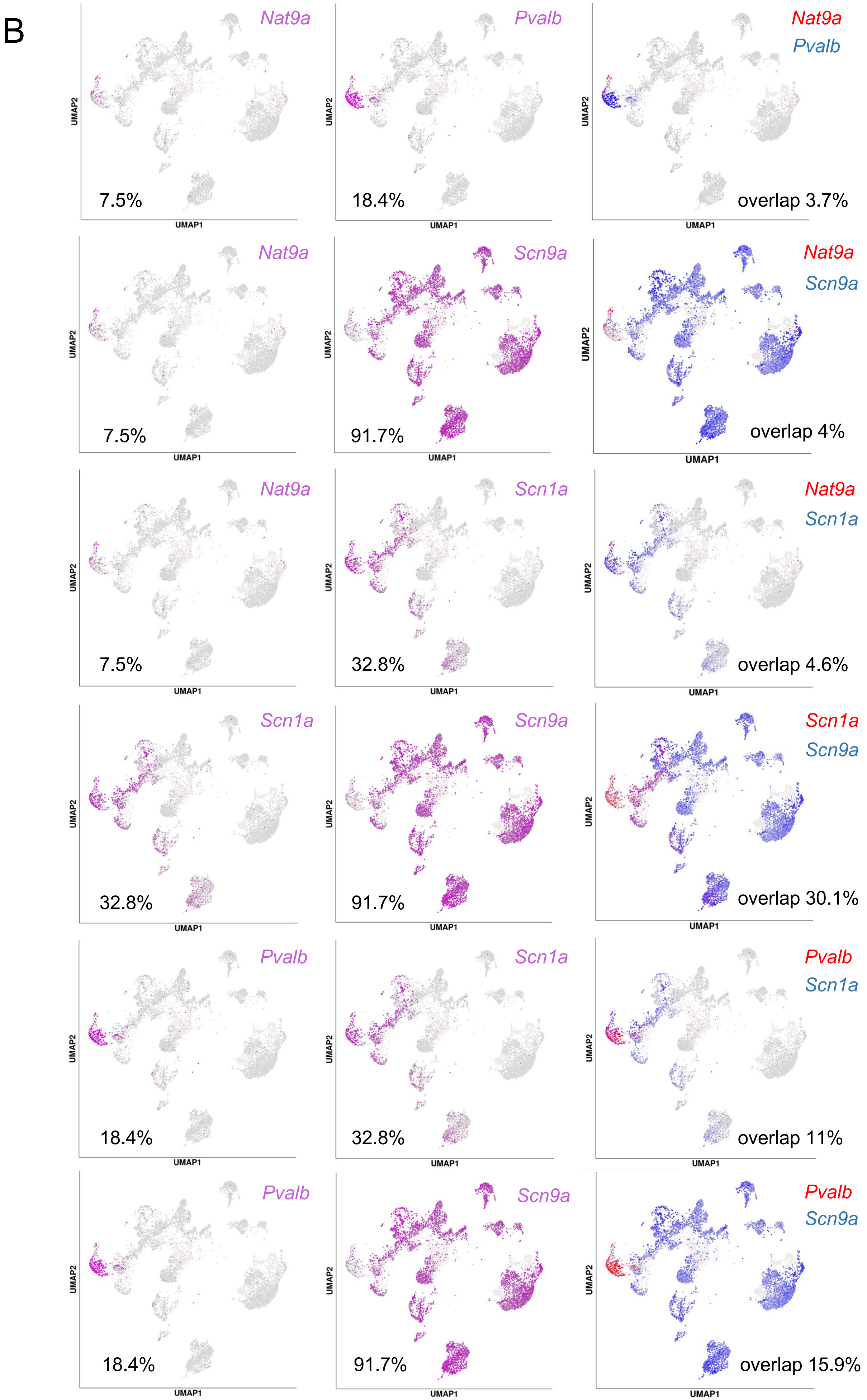
B
Nat9a
7.5%
Pvalb
18.4%
Nat9a
Pvalb
overlap 3.7%
Nat9a
7.5%
Scn9a
91.7%
Nat9a
Scn9a
overlap 4%
Nat9a
7.5%
Scn1a
32.8%
Nat9a
Scn1a
overlap 4.6%
Scn1a
32.8%
Scn9a
91.7%
Scn1a
Scn9a
overlap 30.1%
Pvalb
18.4%
Scn1a
32.8%
Pvalb
Scn1a
overlap 11%
Pvalb
18.4%
Scn9a
91.7%
Pvalb
Scn9a
overlap 15.9%
UMAP1
UMAP2

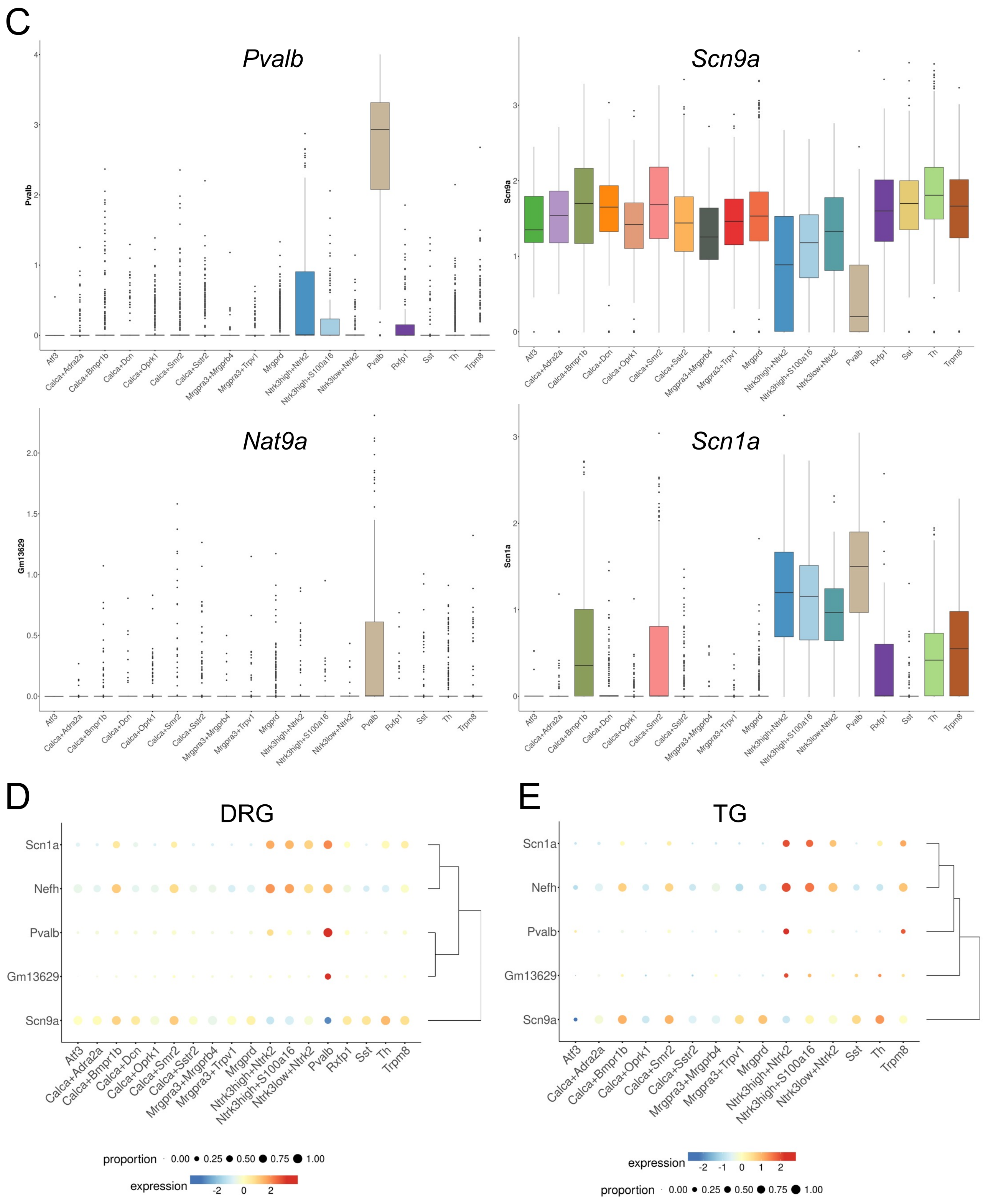

Suppl. Fig 5 C-E: **Single cell transcriptomics analysis.** (**C**) Harmonized mouse DRG RNAseq data [50] were used to compare *Nat9a*, *Scn1a*, *Scn9a* and *Pvalb* RNA expression levels in sub-types of DRG neurons. The data show that *Nat9a* lncRNA is mainly expressed in *Pvalb*+ DRG neurons, which also show high levels of *Scn1a* but low levels of *Scn9a* *m*RNA expression. (**D** and **E**) Bubbleplot (heatmap) of *Nat9a*, *Scn1a*, *Scn9a* and *Pvalb* RNA expression in DRG (**D**) and TG (**E**) neurons show that *Nat9a* (*Gm13629*) is primarily expressed in *Pvalb*+ proprioceptors, which on the other hand show depleted levels of Scn9a compared to other *Scn9a*+ types of cells in both DRG and TG. Data visualisation was made possible by Harmonized DRG and TG Reference Atlas [50].

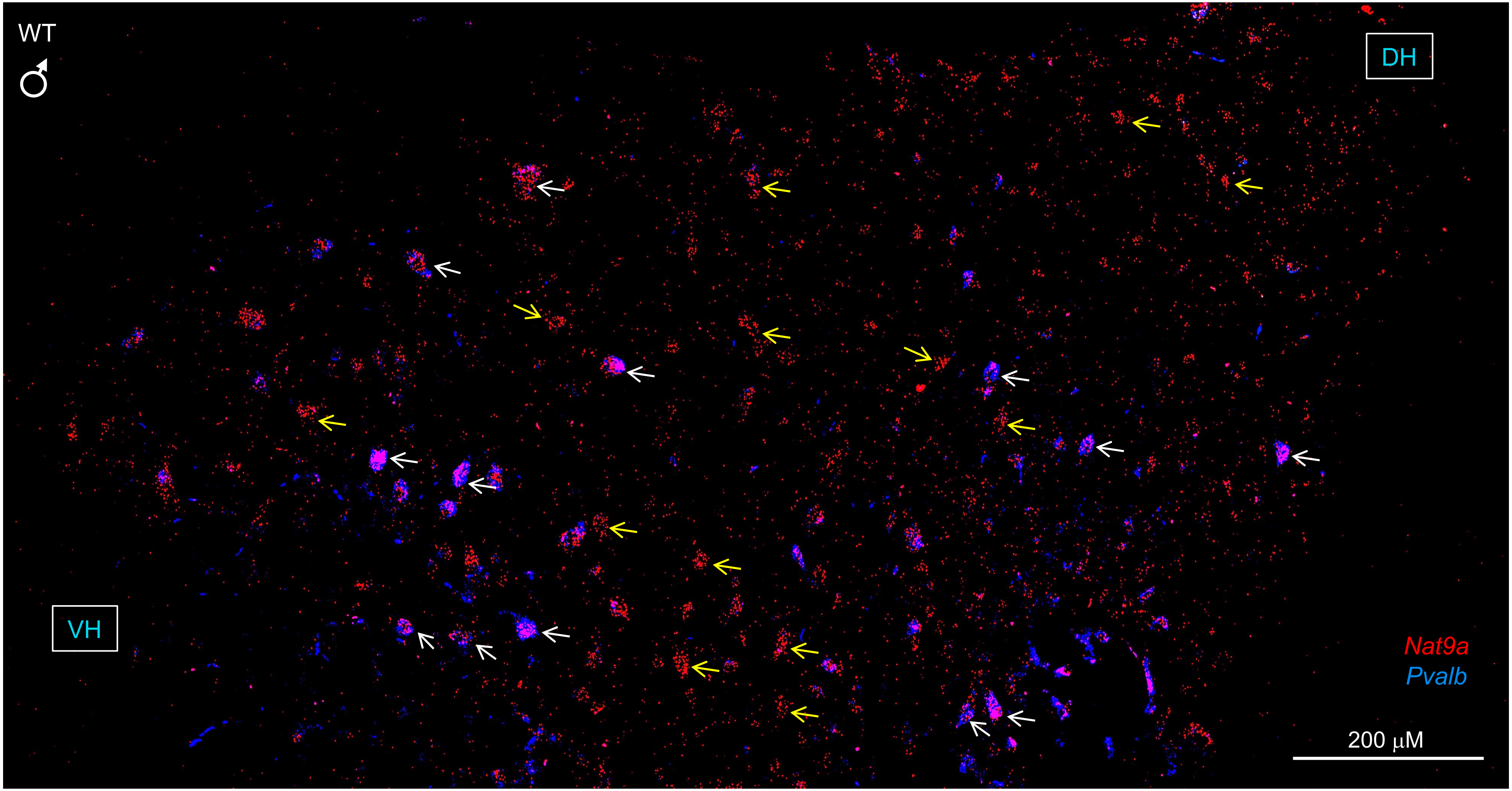

Suppl. Fig S6 ***Nat9a* in *Pvalb+* cells of wild type male mouse spinal cord (Box 1 from Figure 3A).** Localization of *Nat9a* lncRNA (red) was compared to *Pvalb* mRNA (blue). *Nat9a* is expressed at a high level in multiple large neurons (indicate by white arrows), many of which (but not all) are *Pvalb+*. *Nat9a* only expressing large neurons are indicated with yellow arrows. Positions of dorsal horn and ventral horn areas are indicated as DH (top right) and VH (bottom left). Scale bar is in white.

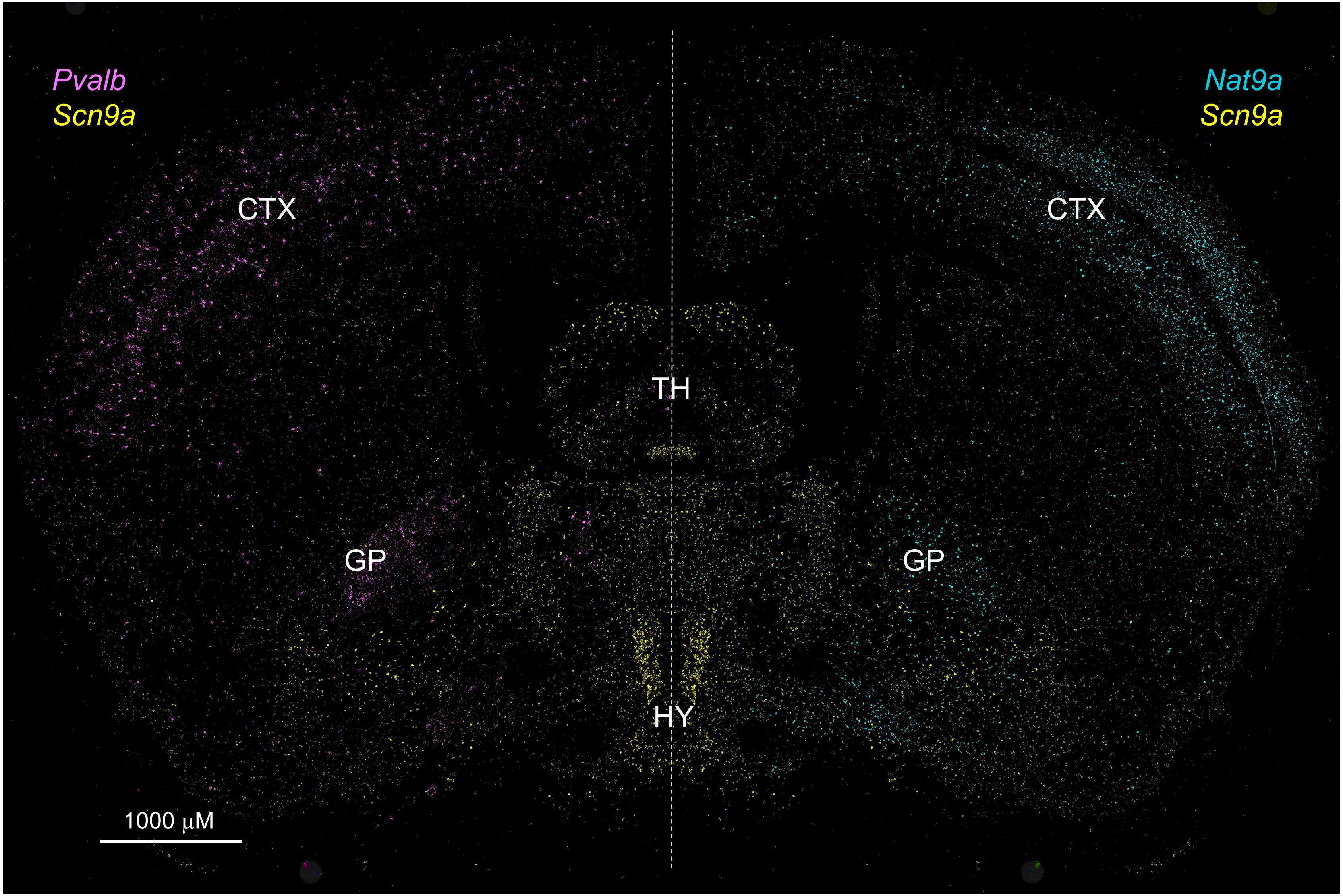

Suppl. Fig S7 ***Nat9a, Scn9a* and *Pvalb* expression patterns in brain.** Localization of *Pvalb* mRNA (in purple) compared to *Scn9a* mRNA (in yellow) on the left side, and comparison of *Nat9a* lncRNA (in cyan) to *Scn9a* mRNA (in yellow) on the right. *Nat9a* lncRNA expression is discordant with that of *Scn9a* mRNA, and similarly *Scn9a* mRNA does not overlap with *Pvalb*+ neurons. *Nat9a* and *Pvalb* expression is enriched in cortex (CTX) and globus pallidus (GP), whereas *Scn9a* mRNA is enriched in thalamus (TH) and hypothalamus (HY) areas. Region 6 sections of mouse brain prepared by Zyagen. Scale bar is in white.

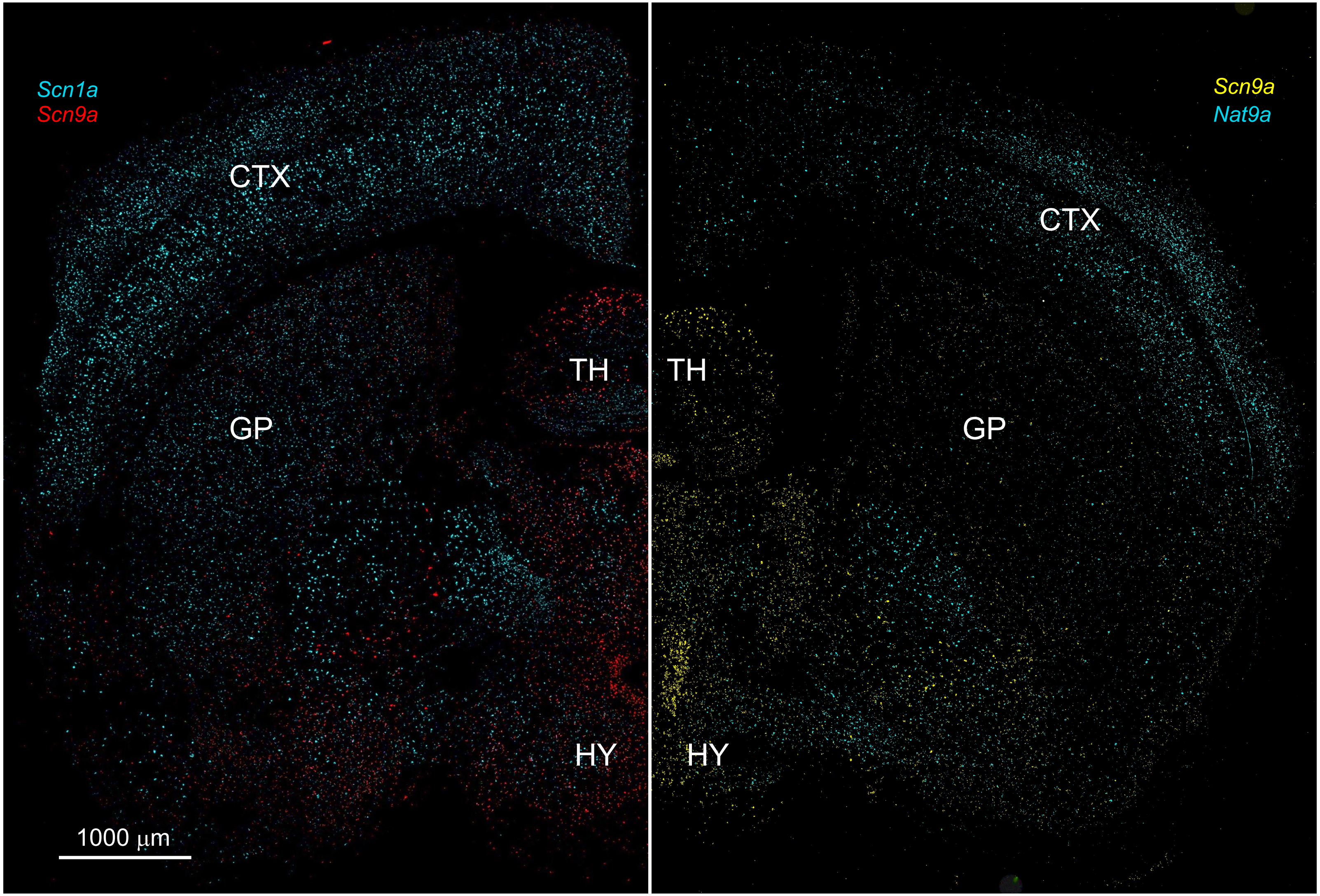

Suppl. Fig S8. *Nat9a, Scn1a* and *Scn9a* expression patterns in brain. Distribution of *Scn1a* mRNA expression (in cyan) compared to *Scn9a* mRNA (in red) on the left panel, and comparison of *Scn9a* mRNA expression (in yellow) to that of *Nat9a* lncRNA (in cyan) on the right panel. *Scn1a* mRNA expression is discordant with that of *Scn9a* mRNA, but mostly overlaps with that of *Nat9a* lncRNA. Both *Nat9a* and *Scn1a* expression is enriched in cortex (CTX), whereas *Scn9a* mRNA is enriched in thalamus (TH) and hypothalamus (HY) areas. Images show data from two independent experiments. Region 6 sections of mouse brain prepared by Zyagen. Scale bar in white.

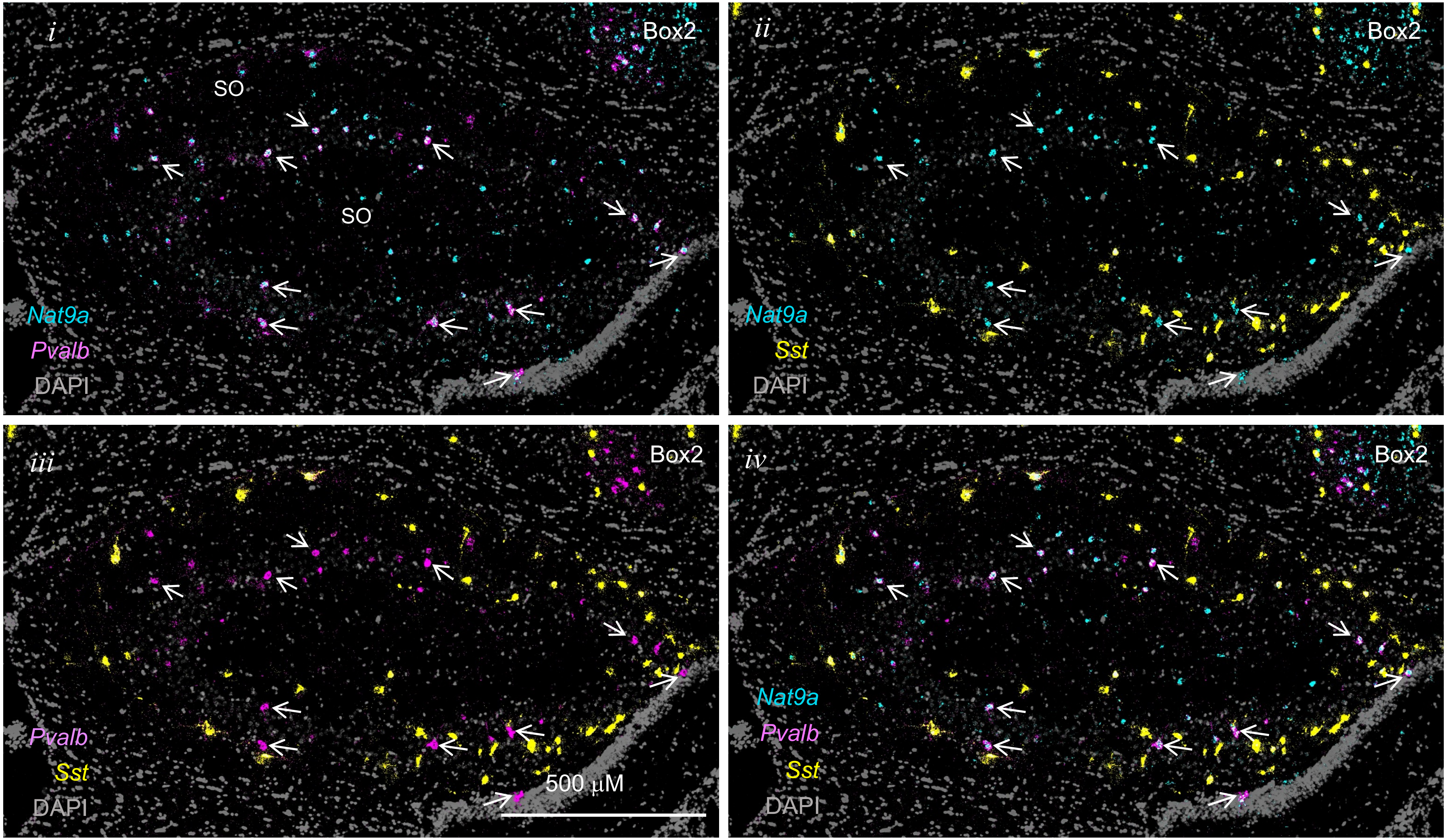

Suppl. Fig S9a *Nat9a*, *Pvalb* and *Sst* expression patterns in hippocampus (Box 2 from Figure 5A).
Expression of *Nat9a* lncRNA (cyan) compared to *Pvalb* mRNA (magenta), and *Sst* mRNA (yellow) expression patterns in medulla of mouse brain. Similar to other areas of brain expression of *Nat9a* lncRNA (cyan) coincides with *Pvalb* mRNA (magenta) resulting in white colour signal, indicated with white arrows, but not with *Sst* mRNA suggesting that like in cortex and other parts of brain only Pvalb+ interneurons express *Nat9a*+ lncRNA. DAPI staining of nuclei is shown in grey. Scale bar is in white.

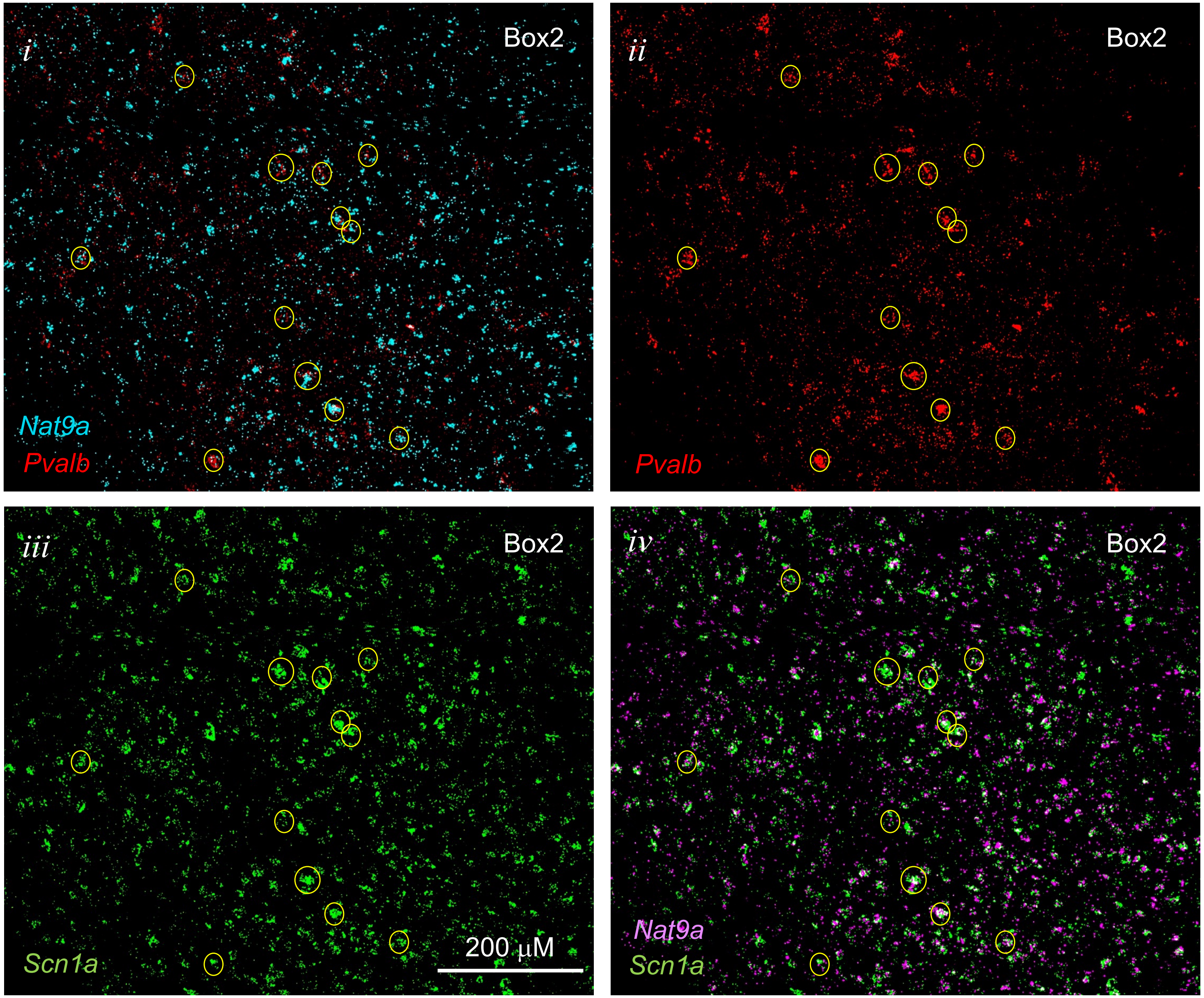

Suppl. Fig S9b *Nat9a, Scn1a* and *Pvalb* expression patterns in medulla (Box 2 from Figure 6A).
Expression of *Nat9a* lncRNA (cyan (*i*) or purple (*iv*)) compared to *Pvalb* mRNA (red) and *Scn1a* mRNA (green) expression patterns in medulla of mouse brain. Similar to other areas of brain, expression of *Nat9a* lncRNA coincides with *Pvalb* mRNA (encircled in yellow, panels ***i*** and ***ii***), and with *Scn1a* mRNA (encircled in yellow, panels ***iii*** and ***iv***), although there is a population of *Nat9a*+ neurons that do not seem to express *Pvalb* and/or *Scn1a*, similar to cortex area.

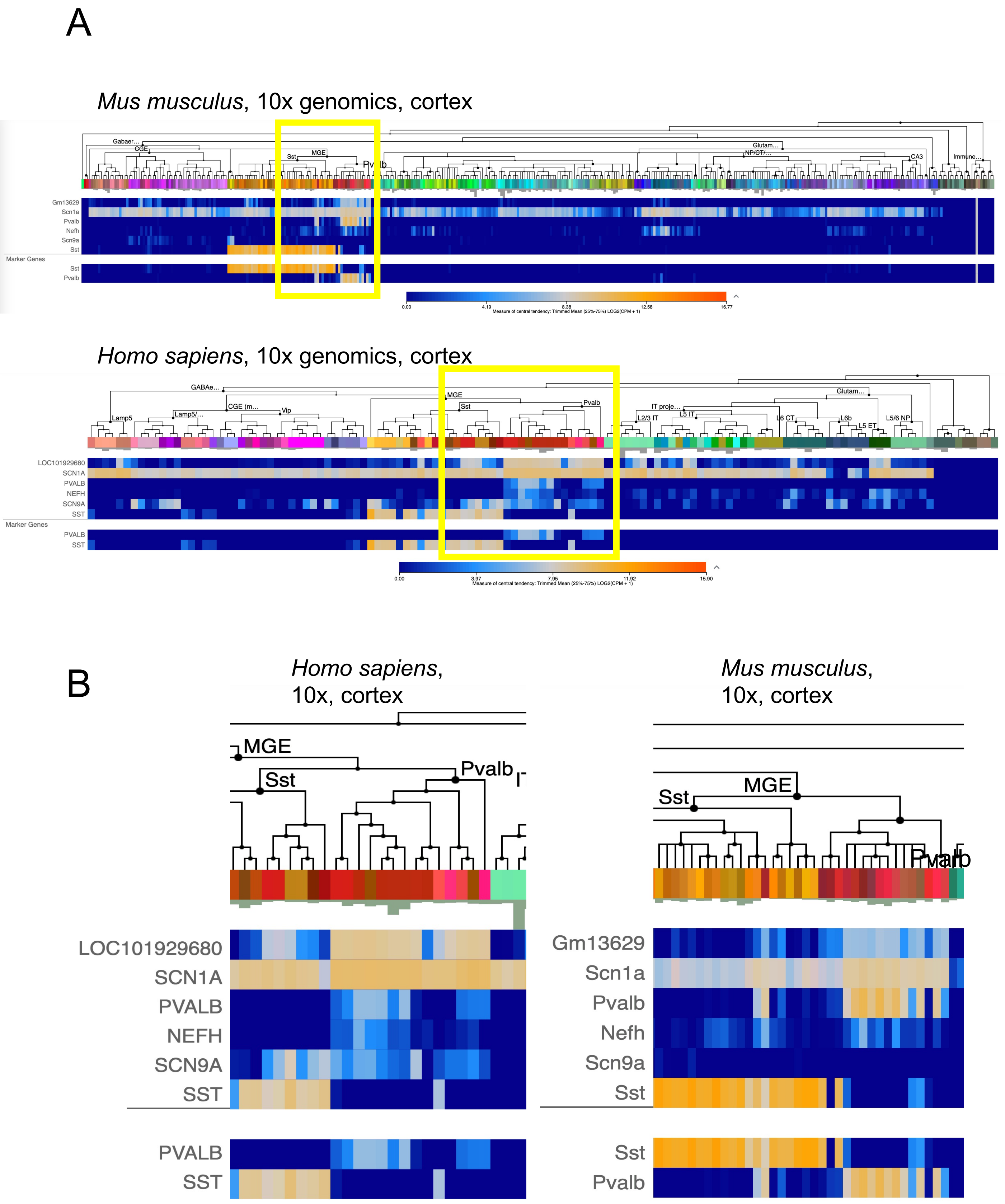

Suppl. Fig S10: **Gene expression patterns in human and mouse cortex neurons.** Allen Brain Map single-cell RNA seq data for human (upper) and mouse (lower) cortex indicating concordant expression of *LOC101929680* (human *NAT9A*) and *Gm13629* (mouse Nat9a) with *SCN1A/Scn1a* and *PVALB/Pvalb* and discordant expression with *SCN9A/Scn9a*. The yellow boxed regions in (**A**) are shown in (**B**).

A

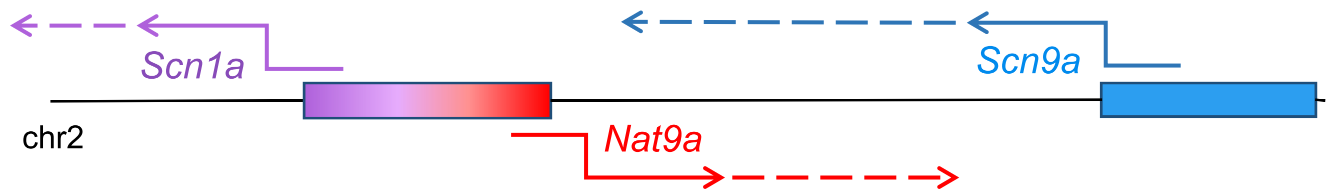

B

Common Transcription Factors

Scn1a / *Nat9a*	*Scn9a*
E2f6	E2f6
Elf3	Elf3
Elf3	Elf3
Elf5	Elf5
Etv5	Etv5
Klf9	Klf9
Pou2f3	Pou2f3
Prdm9	Prdm9
Prdm9	Prdm9
Rara :: Rxrg	Rara :: Rxrg
Znf680	Znf680

PSNF2 specific Transcription Factors

Scn1a	*Nat9a*	*Scn9a*
Elk4	Atf2	
Hlf	E2f6	E2f6
Rora	Etv5	Etv5
Tef	Fos	Esrrg
	HoxD9	FoxJ2
	Hsf2	Foxj3
	Jun	Klf6
	Klf9	Klf9
	Mef2c	Irf1
	Meis2	Stat3
	Meis3	Stat5b
	Prdx1	Tcf4
	Runx1	Zeb1
	Etv1	
	Zbtb18	

Suppl. Fig S11 A, B: **Transcription factors at the *Scn1a/Nat9a* and *Scn9a* promoters.**
(**A**) Schematic of the *Scn1a*, *Nat9a* and *Scn9a* locus (chr2, GRCm39/mm39) showing positions and direction of genes. Shared, divergent promoter area for *Scn1a* and *Nat9a* (*Gm13629*) is shown as one box filled with gradient colour (magenta/red, Chr2: 66,440,750-66,441,200) and *Scn9a* promoter area is shown as a blue box (Chr2: 66,635,950-66,635,350). (**B**) Transcription factors DNA recognition sequences which are present in *Scn1a*, *Nat9a* and *Scn9a* promoter regions are shown in purple (within 200bp upstream of *Scn1a*), red (within 200bp upstream of *Nat9a*) and blue (*Scn9a*). Transcription factors common between *Scn1a/Nat9a* and *Scn9a* promoters are indicated in the left side of the table, with shared transcription factors highlighted in green. Transcription factors that are expressed specifically in PSNF2 proprioceptors (Pvalb+ neurons) in DRG are indicated in the right side of the table, with transcription factors common for both *Scn1a/Nat9a* and *Scn9a* promoters (E2f6, Etv5 and Klf9) highlighted in green.

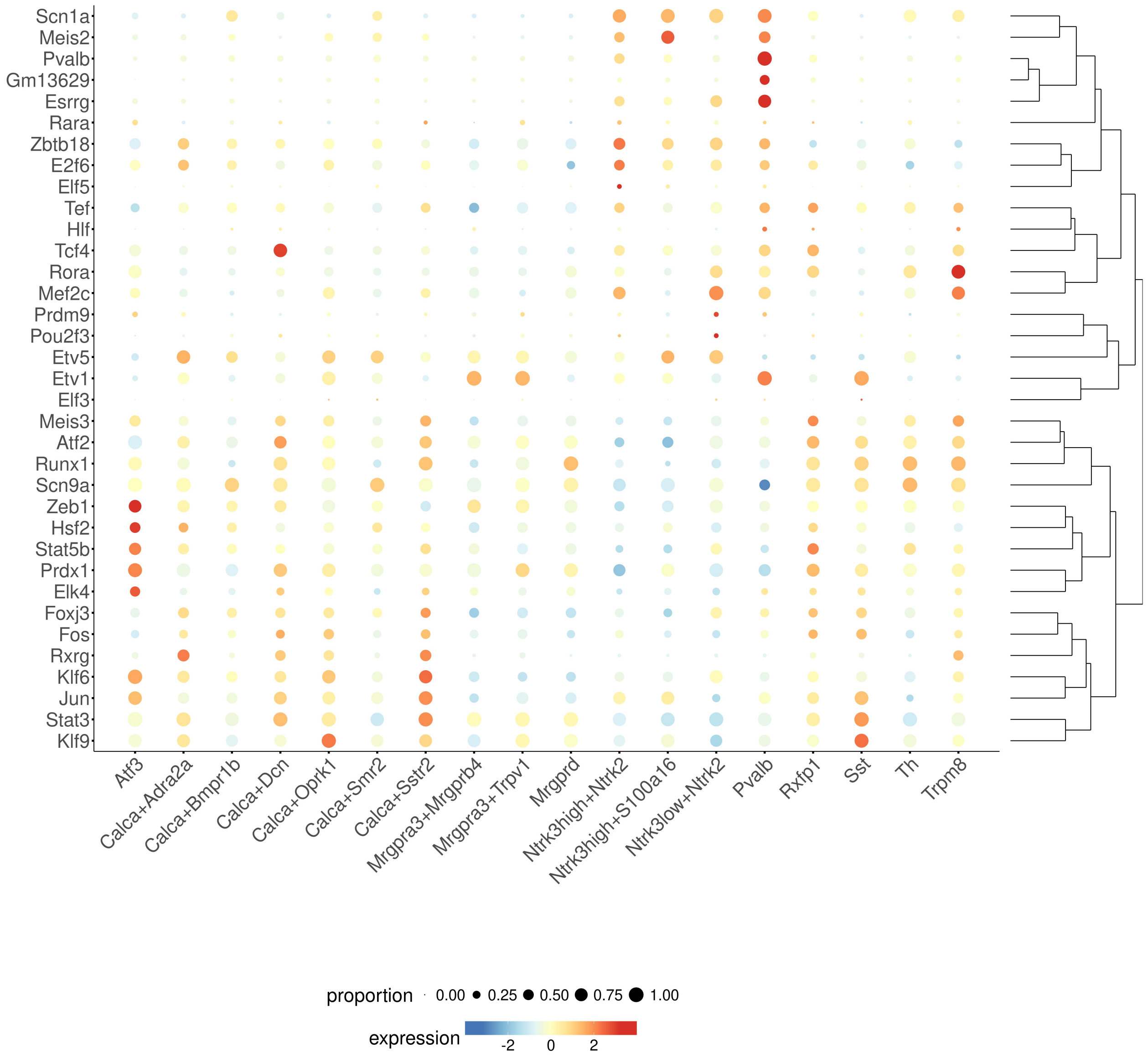

Suppl. Fig 11C: **Single cell transcriptomics analysis.** (**C**) Harmonized mouse DRG RNAseq data [50] were used to compare *Nat9a (Gm13629)*, *Scn1a*, *Scn9a* and *Pvalb* RNA expression in sub-types of DRG neurons with the expression levels of transcription factors which were identified for *Nat9a (Gm13629)*, *Scn1a* and *Scn9a* promoter regions (**Suppl. Fig 11B**). The bubbleplot (heatmap) data show that there are two recognisable populations of transcription factors that are either expressed in *Scn1a*+ and *Pvalb*+ DRG neurons – the upper part of the panel, or those transcription factors that are primarily expressed in *Scn9a*+ DRG neurons – the lower part of the panel. Data visualisation was made possible by Harmonized DRG and TG Reference Atlas [50].
