# Supplementary material for: Natural antisense transcript Nat9a suppresses Scn9a (NaV1.7) expression in parvalbumin-positive proprioceptive and inhibitory neurons
Source: Sci Rep. 2026 Apr 16;16:17733. doi: 10.1038/s41598-026-48500-8 (PMC13247247; doi:10.1038/s41598-026-48500-8)
Supplement: Supplementary file 3 — Supplementary Material 3 [file 41598_2026_48500_MOESM3_ESM.pdf]

Uncropped gel image of figure 1B

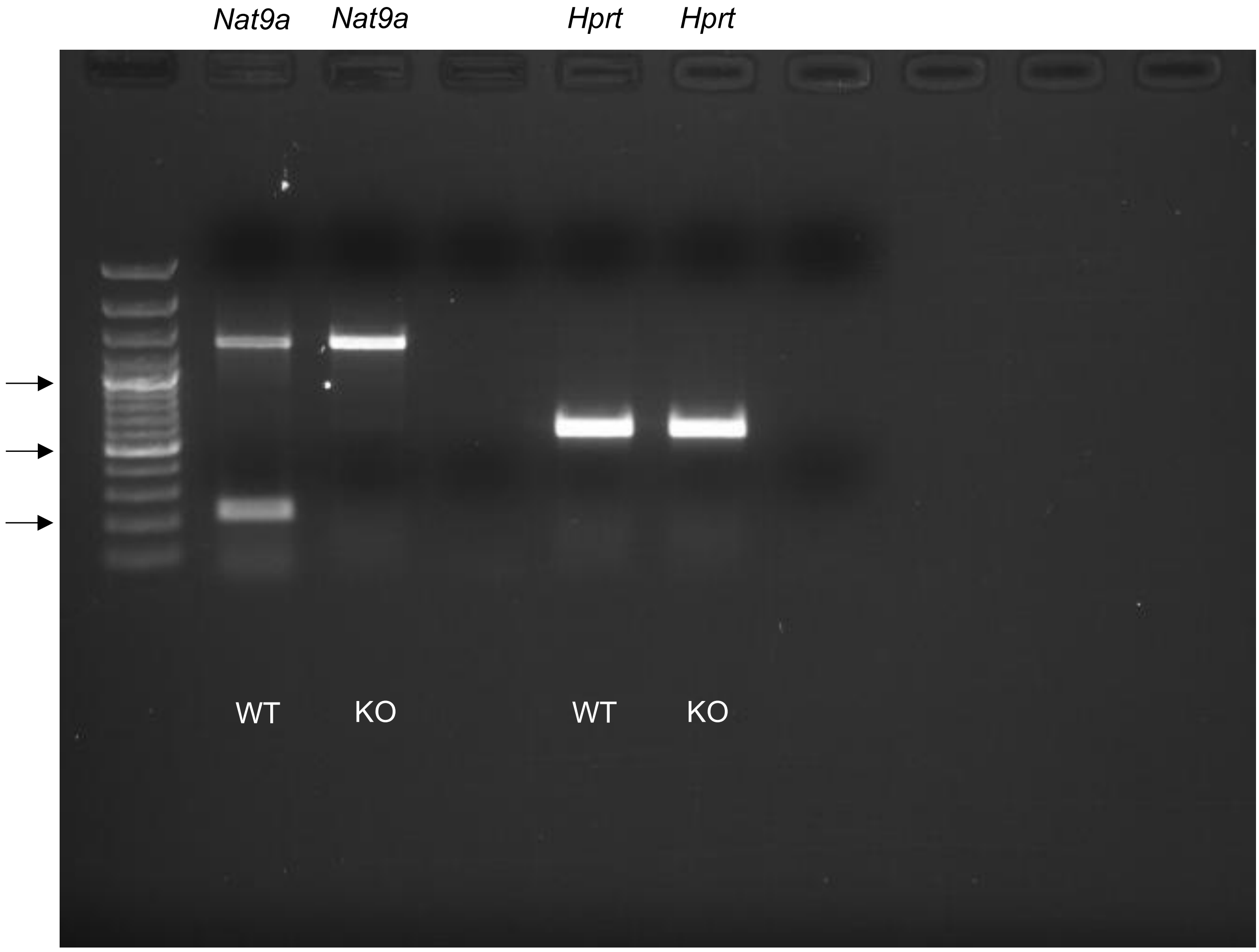


Gel image of RT-PCR amplification of DRG cDNA from *Nat9a* knockout and wild type littermate control. *Nat9a* transcript (222 bp) is amplified in the wild type mouse and no transcript is amplified from knockout mouse DRG (note the upper band corresponding to an expected 1343 bp product is amplified from residual genomic DNA). Housekeeping gene *Hprt* was tested in both genotypes to confirm cDNA was present in the sample (605 bp). Arrows correspond to 200bp (bottom), 500bp (middle) and 1000bp (top).
